# Circular RNA circATP9A promotes non-small cell lung cancer progression by interacting with HuR and by promoting extracellular vesicles-mediated macrophage M2 polarization

**DOI:** 10.1186/s13046-023-02916-6

**Published:** 2023-12-05

**Authors:** Yuanshan Yao, Chunji Chen, Jing Wang, Haojie Xuan, Xiuxiu Chen, Zheng Li, Fuzhi Yang, Bin Wang, Siyun Lin, Saitian Li, Dongfang Tang, Libao Gong, Wen Gao

**Affiliations:** 1 Department of Thoracic Surgery, Shanghai Key Laboratory of Clinical Geriatric Medicine, HuaDong Hospital Affiliated to Fudan University, Shanghai, 200041 China; 2 Department of Abdominal Oncology, The Cancer Center of the Fifth Affiliated Hospital, Sun Yat-Sen University, Zhuhai, 519000 Guangdong Province China

**Keywords:** NSCLC, circATP9A, Extracellular vesicles, HuR, Macrophages

## Abstract

**Background:**

CircRNA is recognized for its significant regulatory function across various cancers. However, its regulatory role in non-small cell lung cancer (NSCLC) is still largely uncharted.

**Methods:**

Analysis based on public databases is completed using R software. circATP9A was identified by two circRNA datasets of NSCLC from the Gene Expression Omnibus database. To examine the impact of circATP9A on the phenotype of NSCLC, we conducted both in vitro and in vivo functional experiments. The mRNA and protein levels of specific molecules were determined through quantitative real-time PCR and western blot assays. RNA pulldown and RNA immunoprecipitation assays were performed to verify the interaction between RNA and protein. The functional role of extracellular vesicles (EVs)-circATP9A on tumor-associated macrophage (TAM) polarization was assessed using co-culture system and cell flow cytometry.

**Results:**

Here, we elucidates the functional role of circATP9A in NSCLC. We demonstrated that circATP9A can foster the progression of NSCLC through in vivo and in vitro experiments. From a mechanistic standpoint, circATP9A can interact with the HuR protein to form an RNA–protein complex, subsequently amplifying the mRNA and protein levels of the target gene NUCKS1. Further, the PI3K/AKT/mTOR signaling was identified as the downstream pathways of circATP9A/HuR/NUCKS1 axis. More notably, hnRNPA2B1 can mediate the incorporation of circATP9A into EVs. Subsequently, these EVs containing circATP9A induce the M2 phenotype of TAMs, thereby facilitating NSCLC development.

**Conclusions:**

Our discoveries indicate that circATP9A could serve as a promising diagnostic indicator and a therapeutic target for NSCLC.

**Supplementary Information:**

The online version contains supplementary material available at 10.1186/s13046-023-02916-6.

## Introduction

Non-small cell lung cancer (NSCLC) is the dominant form of lung cancer, constituting around 85% of all reported lung cancer instances [1]. It is further divided into a variety of subtypes, with adenocarcinoma, squamous cell carcinoma, and large cell carcinoma being the most widespread [2]. Even with progress in early detection and treatment techniques, NSCLC is commonly diagnosed at an advanced stage, greatly reducing treatment alternatives and resulting in a bleak prognosis [3]. The 5-year survival rate for late-stage NSCLC patients remains dishearteningly low, highlighting the urgency for sustained investigation and enhanced treatment modalities. The etiology of NSCLC is multifaceted and involves a combination of genetic and environmental influences [4]. A host of genetic modifications have been found in NSCLC, such as anomalies in the epidermal growth factor receptor (EGFR) and KRAS genes or changes concerning the anaplastic lymphoma kinase (ALK) gene, leading to the advent of targeted therapeutic interventions [5]. Moreover, immunotherapy, specifically immune checkpoint inhibitors targeting the PD-1/PD-L1 axis, has surfaced as an encouraging treatment avenue for NSCLC, conferring significant survival improvements for some patients [6]. Despite this, not all patients react favorably to these treatments, and the development of resistance is a possibility, instigating ongoing research to detect biomarkers that can forecast patient prognosis and formulate tactics to surmount resistance.

Circular RNAs (circRNAs) are a unique subclass of non-coding RNAs distinguished by their covalently closed loop structures, unlike their linear counterparts [7]. Notable for their robust stability, conservation, and often tissue or development-stage-specific expression, circRNAs have recently garnered considerable interest for their implicated roles across various diseases, prominently including cancer [8, 9]. Within the landscape of tumorigenesis, circRNAs demonstrate regulatory capacity at diverse stages—spanning from transcription to translation [10]. They exhibit functionality as microRNA sponges, engage in the formation of RNA–protein complexes through interaction with RNA-binding proteins, modulate transcription of parent genes, and can even give rise to functional peptides through translation [11]. Across various cancer types, a dysregulation of circRNA expression is commonly observed, often with ties to the progression and prognosis of the disease. Certain circRNAs act as oncogenes, fostering tumor growth, metastasis, and resistance to therapy, while others assume the role of tumor suppressors, curbing these processes [12]. Given the multifaceted roles that circRNAs play in the cancer biology, they hold substantial promise as innovative diagnostic biomarkers and therapeutic targets for cancer. For example, Li et al. discovered that circNDUFB2 can break the stability of IGF2BP through the TRIM25/circNDUFB2/IGF2BPs complex, further suppressing NSCLC progression [13]. Xu et al. found that the circRNA hsa_circ_0000326 can enhance lung cancer progression by regulating the miR-338-3p/RAB14 axis [14]. Moreover, Liu et al. found that N6-methyladenosine-modified circIGF2BP3 impairs CD8 + T-cell responses and encourages tumor immune evasion by enhancing the deubiquitination of PD-L1 in NSCLC [15].

In this paper, we have shed light on the significant role that circATP9A plays in NSCLC. We established that circATP9A can drive NSCLC progression. On a mechanistic level, circATP9A engages with the HuR protein, forming an RNA–protein complex and subsequently enhancing both the mRNA and protein expression levels of its target gene, NUCKS1. Importantly, we identified the PI3K/AKT/mTOR signaling pathway as a downstream effector of the circATP9A/HuR/NUCKS1 axis. Furthermore, the protein hnRNPA2B1 has been shown to facilitate the encapsulation of circATP9A into extracellular vesicles (EVs). These EVs, enriched with circATP9A, subsequently induce the M2 phenotype in tumor-associated macrophages (TAMs), thus promoting NSCLC progression. The findings of our study point to circATP9A as a potential diagnostic tool and therapeutic target in NSCLC management.

## Methods

### Samples collection

The open-accessed circRNAs expression profile and corresponding tissue information were obtained from the Gene Expression Omnibus (GEO), GSE112214 and GSE158695 projects. Detailed, the GSE112214 and GSE158695 projects both provide the circRNAs expression profile of three NSCLC and normal adjacent tissues (NATs), whose annotation platform is GPL19978. Eighty patients with NSCLC and normal adjacent tissues (NATs) underwent surgical resection at the Huadong hospital. Totally, 39 NSCLC samples and 20 NATs were collected fromthe Huadong hospital. The samples were promptly preserved in liquid nitrogen at the Huadong hospital. Each sample underwent independent evaluation by two distinct pathologists. Importantly, no patients had been subjected to any preoperative procedures. We also gathered preoperative serum samples from each participant. The Ethical Committee of Huadong hospital, approved the use of these specimens. Written informed consent was procured from every participating patient. Pan-cancer data was get from the UCSC Xena database (https://xenabrowser.net/datapages/). Expression profile and clinical data of NSCLC patients in The Cancer Genome Atlas Program (TCGA) database was downloaded from the TCGA-GDC [16]. Open-accessed clip-seq data was get from the ENCORI database (http://starbase.sysu.edu.cn/).

### Cell lines

The human non-small cell lung cancer (NSCLC) cell lines H522, A549, H1299, H460, the human bronchial epithelium cell line (BEAS-2B),human monocytes cell line THP-1 and mouse Lewis lung carcinoma cell line (LLC), were all obtained from the American Type Culture Collection (ATCC, Manassas, VA). These cell lines were all grown in a controlled environment at 37 °C with a 5% CO_2_ concentration, using RPMI 1640 medium (Biosharp, Guangzhou, China) or DMEM medium (Biosharp, Guangzhou, China). The media were further supplemented with 10% fetal bovine serum (FBS; Gibco, South America).

### Animal experiments

Every animal experiment conducted received approval from the Fudan University (20230304Z). Both subcutaneous xenograft and tail vein lung metastasis models were established using female BALB/c nude mice. For the subcutaneous tumor model, roughly 2 × 10^6^ A549 cells suspended in a 40% Matrigel (BD, San Jose, CA, USA) medium were injected into each mouse's flank. Tumor development was monitored and quantified on a weekly basis, with tumor volume calculated using the formula (length × width^2^/2). After a period of four weeks, the mice were euthanized and tumor weights were recorded. For the tail vein metastasis model, an approximate of 1 × 10^6^ A549 cells were introduced via the tail vein into the nude mice. Four weeks later, all mice were euthanized. Their lungs were imaged and then harvested for ensuing analysis. LLC cells were utilized to establish a tail vein metastasis model in C57/B6 mice. For the exosome treatment experiments, exosomes produced by both control and A549 cells, which overexpressed circATP9A, were administered intravenously into the tail vein of C57/B6 mice. Each injection contained 2 μg of exosomes, and the mice received two injections per week. After a total of five injections, the mice were sacrificed for tissue collection and further assessments. Ki67 expression in tumor tissues from xenografted nude mice was quantitatively assessed using the H score. Tumor samples were first fixed, paraffin-embedded, and sectioned for immunohistochemical (IHC) analysis. These sections were stained with a Ki67-specific antibody, highlighting proliferating cells. The H score for Ki67 was calculated by evaluating both the intensity of staining (graded as 0, 1 + , 2 + , or 3 + for none, weak, moderate, or strong staining, respectively) and the percentage of Ki67-positive cells in the tissue. The final H score, a product of the intensity score and the percentage of positive staining.

### Plasmid construction and cell transfection

For stable circATP9A knockdown, three unique shRNAs targeting circATP9A were engineered into lentiviral vectors, a task completed by GenePharma (Suzhou, China). A nonspecific sh-NC vector was employed as the control. Additionally, a full-length circATP9A lentiviral vector, also synthesized by GenePharma, was employed for circATP9A overexpression, with a control vector lacking any circATP9A sequence used as the reference group. For the generation of stable cell lines, A549 and H1299 cells were infected with respective lentiviruses using 1 µl of Polybrene (5 µg/µl) from GenePharma as an enhancer. After 72 h, the cells were exposed to 5 µg/ml of puromycin in medium for selection. Post a 10-day duration, puromycin-resistant cells were deemed as stably transfected. A similar methodology was applied for siRNAs against HuR and NUCKS1-OE plasmids, both sourced from RiboBio (Guangzhou, China). The transfection of si-RNAs and plasmids was executed using Lipofectamine 3000 (Invitrogen, Carlsbad, CA, USA), abiding by the manufacturer’s instructions. The knockdown or overexpression efficacy of specific molecules was validated through qRT–PCR or western blot assays. The sequences of sh-circATP9As and siRNAs are provided in Table S1.

### Quantitative Real-time PCR (qRT–PCR)

Total RNA was extracted using the TRIzol reagent (Invitrogen, Carlsbad, CA, USA), followed by cDNA conversion via the PrimeScript RT Reagent Kit (Takara, Tokyo, Japan). The ensuing qRT-PCR analysis was carried out utilizing the Sybr green system, with GAPDH functioning as the internal standard. RNA relative expression levels were computed via the 2–ΔΔCT method. Table S2 lists the primer sequences used in this study.

### Rnase R treatment

In summary, RNA was retrieved from A549 and H1299 cells with TRIzol reagent (Invitrogen, Carlsbad, CA, USA) according to the provided instructions. This was accompanied by the addition of Rnase R (1.0 U/μg) (Geenseed, Guangzhou, China) to 500-ng RNA, and incubation for 20 min at 37 °C. For control, a similar amount of RNA was processed without Rnase R under identical conditions. The stability of circATP9A versus linear mRNA-ATP9A was then assessed using qRT-PCR.

### Electrophoresis analysis

For gel electrophoresis, a 1% agarose gel was prepared by heating 50 ml of 1 × TAE buffer with 0.5-g agarose until boiling. After cooling to 70 °C–80 °C, 5 μl of 4S GelRed (Sangon Biotech, Shanghai, China) was thoroughly mixed in. After solidification of the poured solution in a mold, the gel was placed in an electrophoresis tank filled with 1 × TAE buffer. Subsequently, 10 μl of DNA samples, mixed with loading buffer, were loaded into each well. The electrophoresis ran at 120 V for 30 min, and the resulting bands on the gel were captured using an ultraviolet imaging system.

### In situ hybridization (ISH)

For ISH, we used a probe targeting the circATP9A splicing site, tagged with 5′-digoxin (DIG) and 3′-DIG. This probe was custom made by Servicebio (Wuhan, China). Scramble probe (negative) and a U6 probe (internal) served as controls. The procedure started with deparaffinization and rehydration of paraffin sections with xylene and a series of graded ethanol. Subsequently, a 20-min incubation with proteinase K at 37 °C was performed, followed by a 10-min Triton-X100 treatment at 4 °C. The sections were then incubated overnight at 37 °C in a hybridization buffer containing the circATP9A probe, and another overnight incubation with the anti-digoxin antibody at 4 °C followed. Afterwards, sections were treated with 5-Bromo-4-Chloro-3-Indolylphosphate/Nitroblue Tetrazolium (BCIP0/NBT) (Beyotime, Shanghai, China) at room temperature for 30 min, and with Nuclear fast red (Servicebio, Wuhan, China) at room temperature for 3 min. Visualization and imaging were done with an Olympus microscope (Olympus, Tokyo, Japan). The H-score for circATP9A was computed as: H-score = Σ (P × I), where P signifies the percentage of stained cells, and I represents the staining intensity score: 0 for no staining, 1 for weak, 2 for moderate, and 3 for intense staining. Table S3 provides details of the ISH probes.

### RNA fluorescence in situ hybridization (FISH)

To ascertain the subcellular localization of circATP9A in NSCLC cells, we used a Cy3-labeled circATP9A-specific probe from RiboBio (Guangzhou, China). Briefly, NSCLC cells (A549 and H1299) were trypsinized and resuspended in medium. Approximately 2000 cells were seeded on a 48-well plate with a glass cover slip. Upon reaching 70% to 90% confluence, cells were thrice washed with PBS and fixed with 3.7% paraformaldehyde. Cell permeabilization was achieved using 0.5% Triton-100 for 10 min at 4 °C. Cells were then pre-hybridized at 37 °C for 30 min, followed by overnight incubation at 37 °C with the circATP9A-FISH probe in a dark hybridization buffer. After hybridization, cells were washed using SSC solutions and stained with DAPI for 15 min in darkness. The prepared slides were observed and photographed using a confocal fluorescence microscope (Carl Zeiss AG, Jenna, Germany). Table S3 contains information about the circATP9A-FISH probe utilized in this experiment.

### Colocalization of circATP9A with HuR

Fluorescence staining was employed to assess the colocalization of circATP9A and HuR in NSCLC cells. Briefly, around 2,000 NSCLC cells were seeded onto a 48-well plate with cover glass. Upon reaching 70%–90% confluence, the cells were washed three times with PBS, then fixed with 3.7% paraformaldehyde. Subsequently, cells were permeabilized using 0.5% Triton-100 for 10 min at 4 °C. Pre-hybridization for 30 min at 37 °C using pre-hybridization buffer was carried out, followed by an overnight incubation at 37 °C with a Cy3-labeled circATP9A-FISH probe (RiboBio) in hybridization buffer in a dark environment. The cells were then permeabilized again with 0.5% Triton-100 for 10 min at 4 °C, before incubating with an anti-HuR antibody (Abcam) overnight at 4 °C in darkness with gentle rotation. Following this, cells were washed with PBS and nuclei were stained with DAPI for 15 min. Finally, cells were visualized and imaged with a confocal fluorescence microscope (Carl Zeiss AG, Jenna, Germany).

### Western blotting

The Total Protein Extraction Kit (KeyGEN, Nanjing, China) was used to extract the total protein in NSCLC cells, and the concentrations were measured by a BCA protein assay kit (KeyGEN, Nanjing, China). Antibodies against HuR (ab200342, 1:1000), NUCKS1 (12,023–2-AP, 1:1000), β-actin (20,536–1-AP, 1:5000), p-AKT (Ser273) (66,444–1-Ig, 1:5000), AKT (60,203–2-Ig, 1:5000), p-mTOR (Ser2448) (67,778–1-Ig, 1:2000), mTOR (66,888–1-Ig, 1:5000), CD9 (20,597–1-AP, 1:1000), CD63 (25,682–1-AP, 1:1000), CD81 (66,866–1-Ig,1:2000), Calnexin (10,427–2-AP, 1:5000), hnRNPA2B1 (14,813–1-AP, 1:2000) were obtained from Abcam and Proteintech. Chemiluminescent signals were detected using Western ECL Substrate (Advansta, Menlo Park, CA, USA) and images were captured with a ChemiDoc Imaging System (Bio-Rad, Hercules, CA, USA).

### RNA pull-down assay

The interactions between circATP9A and HuR, as well as circATP9A and hnRNPA2B1, were verified using biotin-coupled circATP9A probes and control probes, furnished by GenePharma (Suzhou, China). The protocol is outlined as follows: Roughly 1 × 10^7^ A549 and H1299 cells were lysed and sonicated in a 4 °C water bath for half an hour. A fraction (20 µl) of the lysate was allocated for RNA input, while a major portion (80 µl) was set aside for protein input. Probes were then incorporated into the lysate and stirred at room temperature for 16–24 h. Next, 100 µl of streptavidin magnetic beads (MCE, Monmouth Junction, NJ, USA) were added to the lysate and rotated at room temperature for 2–4 h. Using a magnetic stand, the beads were gathered and washed five times with washing buffer (containing PMSF, Protease inhibitor, and Rnase inhibitor). The cleansed beads were resuspended in 1 ml of washing buffer, with 100 µl of this solution used for RNA purification and the remaining 900 µl allocated for protein purification. For RNA extraction, the 100-µl sample was combined with 5 µl of proteinase K (Sangon Biotech, Shanghai, China) and RNA PK buffer, then gently rotated at 50 °C for 45 min followed by 10-min heating at 95 °C to break the formaldehyde cross-links. The RNA was subsequently purified using TRIzol reagent (Invitrogen, Carlsbad, CA, USA), converted into cDNA, and stored at -80 °C for future use. For protein extraction, 300 µl of 4 × loading buffer was added to the remaining 900-µl sample, followed by a 10-min incubation at 100 °C. The supernatant with the protein extract was then isolated using the magnetic stand, and the protein was deployed for mass spectrometry (MS) analysis and Western blotting. The sequences of the circATP9A probe and the control probe are presented in Table S3.

### Silver staining

Overall, proteins extracted from the circATP9A RNA pull-down assay were differentiated using a 10% SDS-PAGE gel. The gel was subsequently stained with a Silver Stain kit (BL620A, Biosharp, Beijing, China) in accordance with the producer’s instructions.

### RNA immunoprecipitation (RIP) assay

For verification of the link between HuR and circATP9A, and hnRNPA2B1 and circATP9A, a RIP assay was conducted employing a RIP kit (Millipore, MA, USA). In essence, approximately 2 × 10^7 A549 and H1299 cells were harvested and lysed with RIP lysis buffer. Interacting RNAs were then precipitated with anti-HuR antibody (ab200342, Abcam) and anti-hnRNPA2B1 antibody (14,813–1-AP, Proteintech). The anti-IgG antibody (ab172730, Abcam) was utilized as a negative control. The co-precipitated RNAs were then isolated using TRIzol reagent (Invitrogen, Carlsbad, CA, USA) and their abundance was evaluated by qRT-PCR.

### Co‐culturing system

To mimic the EV-mediated intercellular communication that takes place between tumor cells and TAMs, an in vitro indirect co-culture model was established. Macrophages and LUAD cells were individually planted into the upper and lower chambers of a Corning® Transwell® cell culture insert (4 μm pore size, Corning Inc., Corning, NY, USA), incorporating a polycarbonate membrane. After a 48-h co-culture period, the cells were harvested for further experiments.

### Isolation Evs from cell medium

Evs were derived from NSCLC cell media through a series of steps: NSCLC cells were cultured in a medium enriched with 10% EV-free FBS. Following a 72-h incubation at 37 °C with 5% CO_2_, the medium was collected and centrifuged under various conditions: initially at 2,000 g for 10 min, then at 3,500 g for 20 min, followed by 10,000 g for 1 h, and finally at 120,000 g for 2 h. All centrifugation phases were carried out at 4 °C. The resultant purified Evs were re-suspended in PBS and then stored at -80 °C for subsequent use.

### Macrophage induction from monocytes and flow cytometry

THP1 cells, cultured in six-well plates, were treated with 100 ng/mL of phorbol-12-myristate-13-acetate (PMA; Sigma-Aldrich) and incubated for 24 to 48 h. After incubation, the medium was swapped with fresh PMA-free medium, and the cells were maintained for another 3 days before use. For the detection of macrophage surface markers, cells in chilled PBS were treated with either anti‐CD206 or anti‐HLA-DR antibodies (both from eBioscience) at 4℃ for 30 min. Following incubation, the cells were washed and subsequently analyzed using a BD Accuri™ C6 flow cytometer (BD Biosciences, San Jose, CA, USA) to detect macrophage surface markers CD206 and HLA-DR.

### Evs internalization

Evs derived from RCC cells were labeled using the PKH26 Red Fluorescent Cell Linker Kit (Umibio, Shanghai, China). Following this, PMA-stimulated THP-1 cells [THP-1 (Mφ)] were co-incubated with these PKH26-tagged Evs in a dark setting overnight. After staining the cells with 4′,6-diamidino-2-phenylindole (DAPI), the EV uptake process was visualized using a confocal fluorescence microscope (Carl Zeiss AG, Jenna, Germany).

### CCK-8 assay, EdU assay, colony formation assay, and transwell assay

The proliferation of NSCLC cells was gauged using the CCK-8 assay, the 5-ethynyl2′deoxyuridine (EdU) assay, and the colony formation assay. The cell invasion and migration capabilities were assessed through transwell invasion and migration assays, executed following the protocols delineated in our previous research [17].

### Statistics analysis

Statistical evaluations were conducted using GraphPad Prism and SPSS Software. A two-tailed Student’s t-test, ANOVA followed by Tukey’s multiple comparisons post-test, and Pearson’s correlation analysis were the methods used for statistical comparisons. All statistical data are expressed as mean ± standard error of the mean. All p values were calculated using a two-sided test, with *p* values < 0.05 considered statistically significant. Each experimental procedure was performed at least three times for reliability. The survival difference in different groups was compared using the Kaplan–Meier method.

## Results

### Identification of circRNAs in NSCLC

**Fig. 1:**
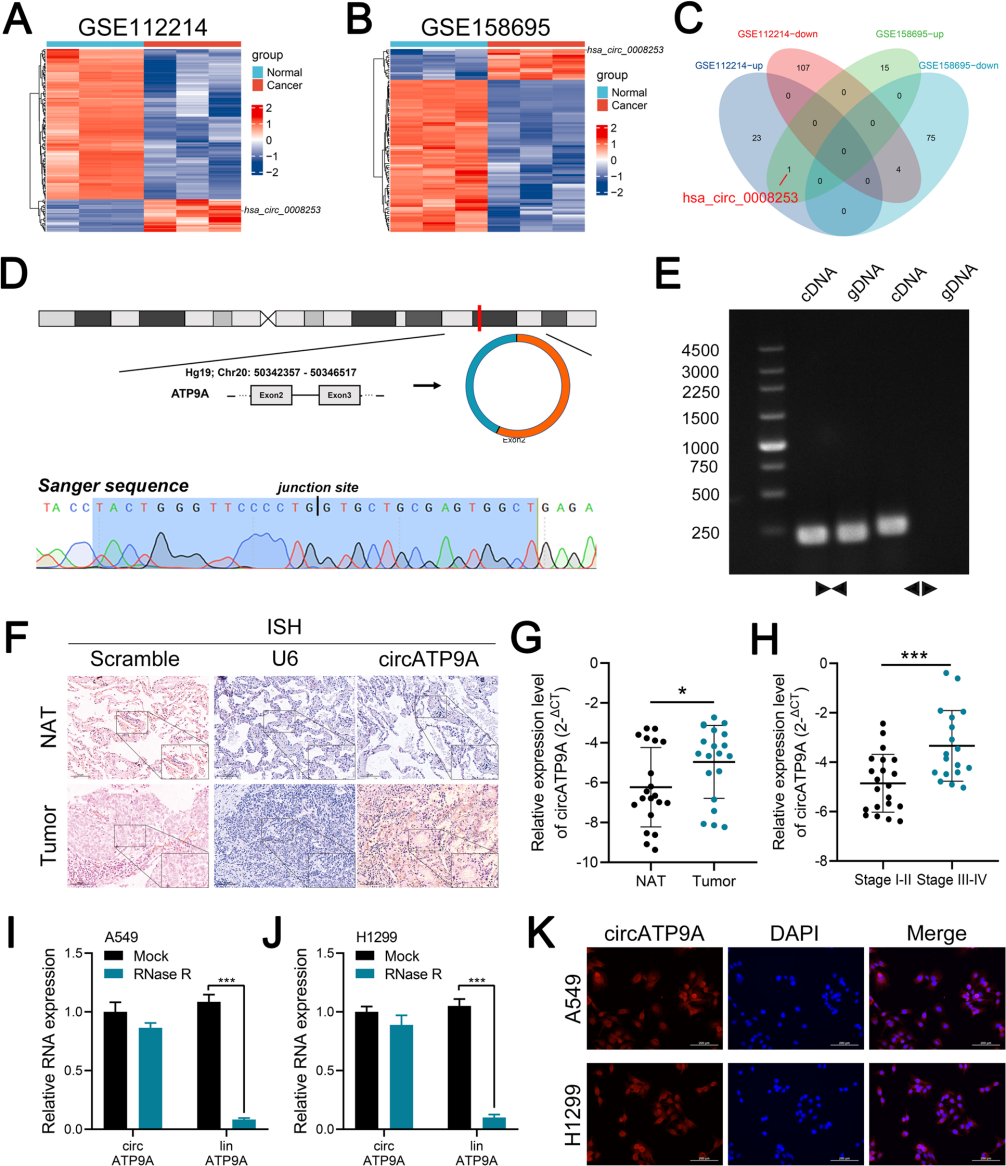
circATP9A was upregulated in NSCLC tissues and associated with poor clinical features. Notes: **A** The cluster heat maps showed the differentially expressed circRNAs in NSCLC tissues and paired NATs from GSE112214; **B** The cluster heat maps showed the differentially expressed circRNAs in NSCLC tissues and paired NATs from GSE158695; **C** The overlapping analysis of upregulated circRNAs in GSE112214 and GSE158695; **D** Schematic illustration showed the circularization of ATP9A exons 2 and 3 to form circATP9A. The back-splicing junction of circATP9A was verified by RT-PCR and Sanger sequencing; **E** circATP9A expression in A549 cells verified by RT-PCR. Agarose gel electrophoresis showed that divergent primers amplified circATP9A in cDNA but not gDNA; **F** Representative ISH images from scramble probe (red: negative control), U6 probe (blue: positive control) and circATP9A probe in NSCLC tissues and paired NATs; **G** The expression level of circATP9A in NSCLC tissues and paired normal adjacent tissues (NATs) by qRT-PCR; **H** The expression level of circATP9A in NSCLC patients stratified by stage; **I**,** J** qRT-PCR detected the expression of circATP9A and linATP9A in NSCLC cell lines with or without RNase R or actinomycin D treatment; **K** FISH assay identifying the subcellular location of circATP9A in the A549 and H1299 cell lines

To identify pivotal circRNAs in NSCLC, we concurrently analyzed two circRNA microarray datasets of NSCLC (GSE112214 and GSE158695). In GSE112214, we found 24 circRNAs upregulated and 111 circRNAs downregulated in tumor tissues relative to their paired normal adjacent tissues (NATs) (|log2 FC|≥ 0.9 and *P* < 0.05) (Fig. 1A). Similarly, in GSE158695, 16 circRNAs were upregulated, and 79 circRNAs were downregulated in tumor samples compared to NATs (|log2 FC|≥ 0.9 and *P* < 0.05) (Fig. 1B). Among these, we observed a consistent upregulation of circRNA I_circ_0008253 across both datasets (Fig. 1C). We renamIhsa_circ_00082 “3 as "cir”ATP9A" since it is derived from the ATP9A gene. circATP9A originates from exon-2 and exon-3 of the ATP9A transcript (chr20:50,342,357–50346517) (Fig. 1D). Sanger sequencing was performed to validate the full-length sequence of circATP9A in A549 cells, revealing consistency with the sequence and backsplicing junction site reported in Circbase (Fig. 1D). To verify the circular nature of circATP9A, we designed convergent and divergent primers to amplify circATP9A in cDNA and genomic DNA (gDNA) via reverse transcription PCR (RT-PCR). Gel electrophoresis results suggested that circATP9A could be amplified by both types of primers in cDNA, but only by convergent primers in gDNA (Fig. 1E). In conclusion, these findings confirm that circATP9A exists in a unique circular form, demonstrating more stability than its linear equivalent.

### circATP9A is highly expressed in NSCLC and is positively correlated with NSCLC progression

ISH analysis pointed out a substantial upregulation of circATP9A in NSCLC tissues, with sparse detection in paired NATs (Fig. 1F). To determine cir’ATP9A's clinical significance in NSCLC, we used qRT-PCR to assess its expression in 20 NSCLC and corresponding NAT samples. The data exhibited a noticeable increase of circATP9A in NSCLC tissues relative to their NATs (Fig. 1G). Furthermore, advanced tumor stages (Stage III-IV) were associated with elevated levels of circATP9A (Fig. 1H). RNase R digestion assays also revealed that circATP9A is more resilient than its linear counterpart, ATP9A mRNA (Fig. 1I-J and Figure S1A). Using FISH assays for sub-cellular localization, circATP9A was found to be primarily concentrated in the cytoplasm of A549 and H1299 cells (Fig. 1K).

### circATP9A promotes the proliferation, invasion, and migration of NSCLC cells

**Fig. 2:**
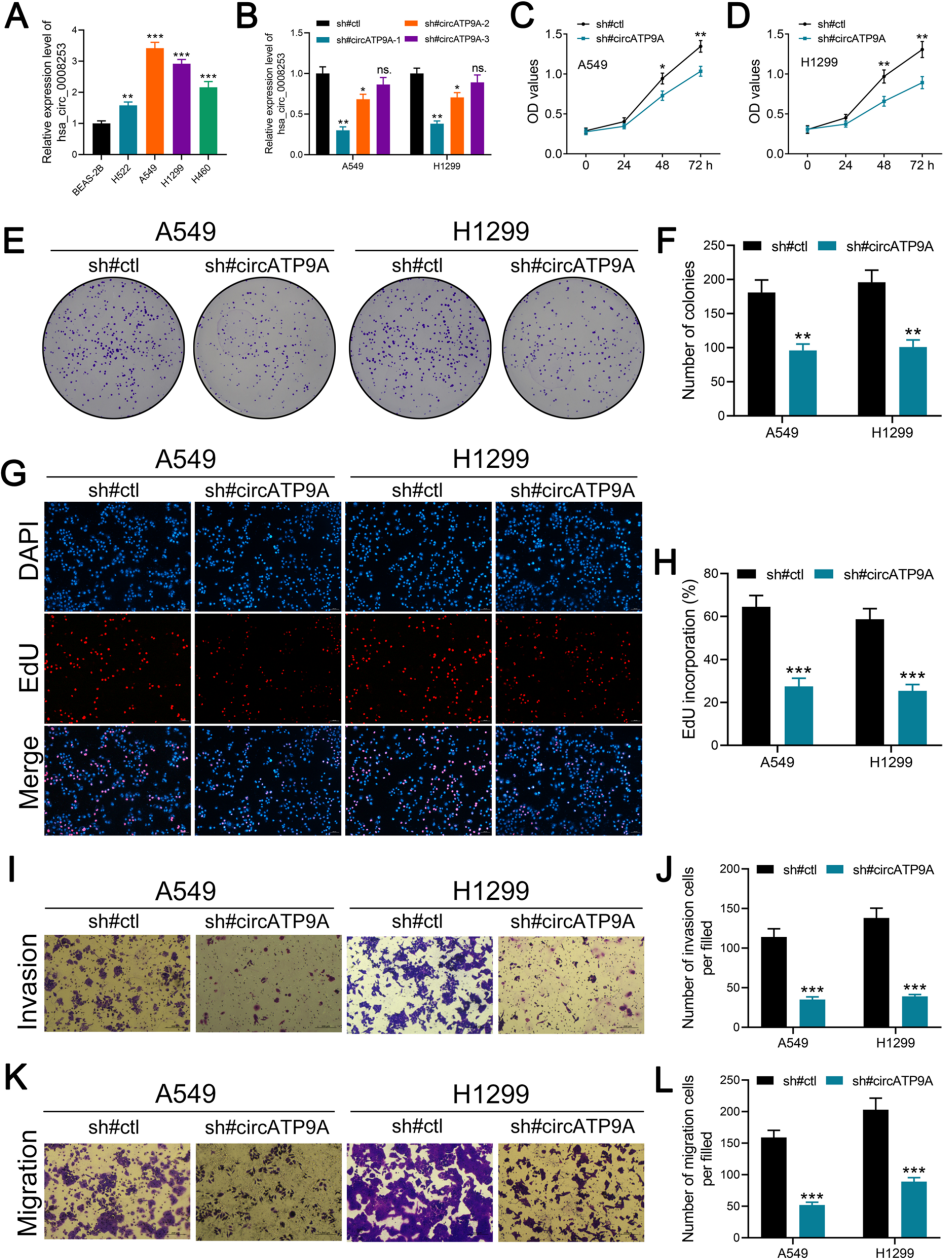
circATP9A promotes proliferation, migration, and invasion of NSCLC cells. Notes: **A** Expression level of circATP9A in NSCLC cells measured by qRT-PCR; **B** The knockdown efficiency of circATP9A in NSCLC cells measured by by qRT-PCR; **C-D** The proliferation ability of A549 and H1299 cells was assessed by CCK8 assay (circATP9A knockdown and control cells); **E**,** F** The proliferation ability of A549 and H1299 cells was assessed by colony formation assay (circATP9A knockdown and control cells); **G**,** H** The proliferation ability of A549 and H1299 cells was assessed by EdU assay (circATP9A knockdown and control cells); **I**, **J** The invasion ability of A549 and H1299 cells was assessed by transwell assay (circATP9A knockdown and control cells); **K**,** L** The migration ability of A549 and H1299 cells was assessed by transwell assay (circATP9A knockdown and control cells)

To investigate circATP9A expression across NSCLC cell lines, we conducted qRT-PCR on four different NSCLC cell lines. circATP9A was significantly more abundant in these lines than in BEAS-2B cells (Fig. 2A), leading us to choose two NSCLC cell lines (A549 and H1299) with relatively high circATP9A expression for further study. We then designed three sh-RNAs to suppress circATP9A and an overexpression vector for circATP9A enhancement. Results demonstrated that sh-RNAs #1 and #2 effectively suppressed circATP9A, whereas the overexpression vector increased its expression (Fig. 2B and Figure S1B). Interestingly, neither the upregulation nor downregulation of circATP9A affected ATP9A mRNA levels (Figure S1C-D). We also observed that circATP9A silencing significantly hindered the growth, migration, and invasion capabilities of A549 and H1299 cells (Fig. 2C-L). The same trend was confirmed in overexpression studies (Figure S2).

### circATP9A promotes the growth and metastasis of NSCLC cells in vivo

**Fig. 3:**
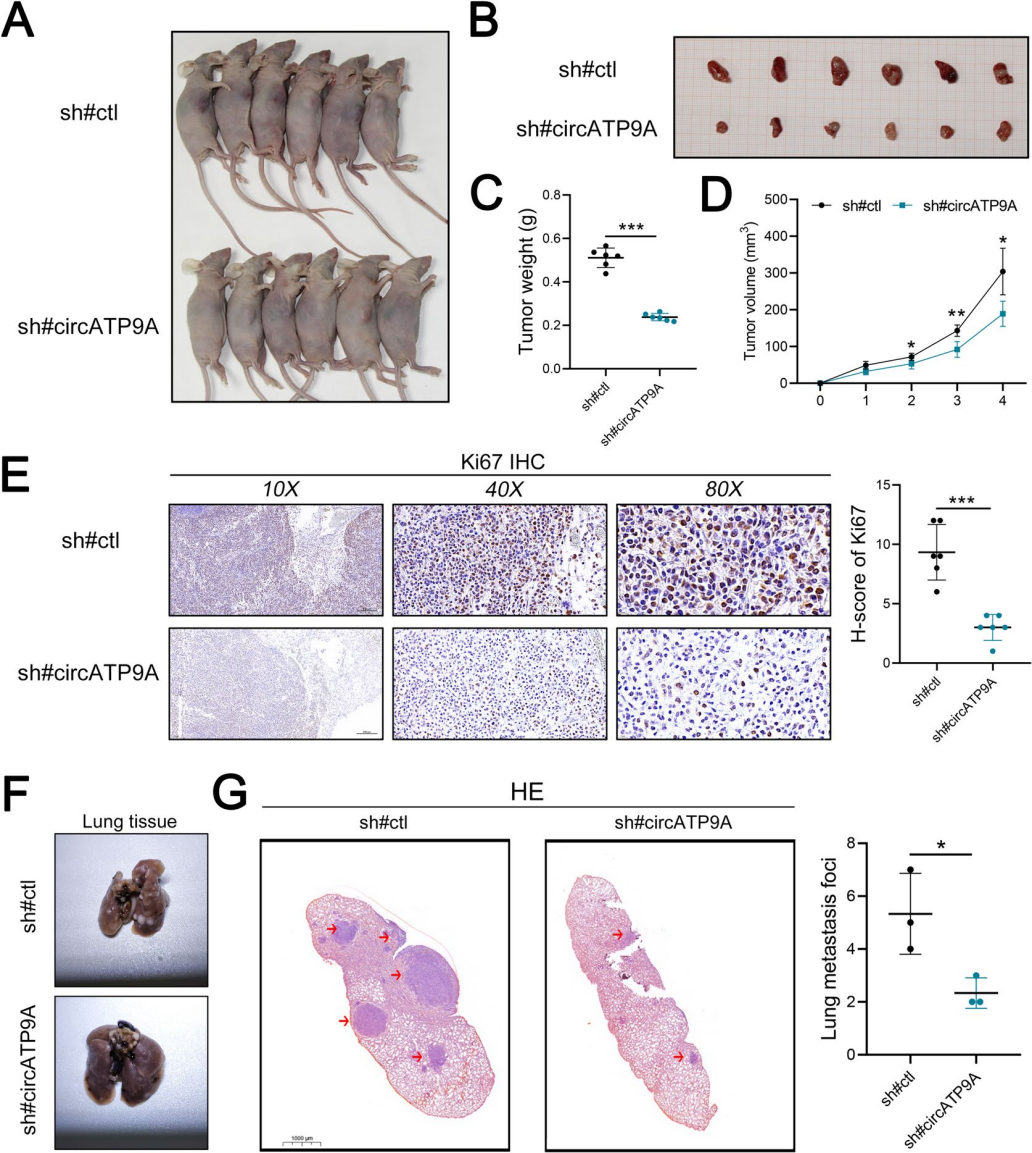
circATP9A promotes the growth and metastasis of NSCLC cells in vivo. Notes: **A-B** Images of xenograft tumors after injection of A549 cells transfected with sh-NC and sh-circATP9A (*n* = 6/group); **C** The subcutaneous tumor weights were weighed at the endpoint time of the experiment; **D** The volumes of subcutaneous tumors were recorded once a week for four consecutive weeks; **E** Ki67 staining of subcutaneous tumor; **F** Images and graph of lung metastasis model after injection of A549 cells transfected with sh-NC and sh-circATP9A; **G** The HE image of mice lung tissue

To further validate the oncogenic role of circATP9A in NSCLC, the subcutaneous xenograft model and lung metastasis model were established. For the subcutaneous xenograft model, the nude mice were randomly divided into two groups (*n* = 6/group). Then A549 cells with stable expression of sh-circATP9A and the control (sh-NC) were injected into the flank of each mouse. The results showed that the tumor growth was remarkably inhibited in the sh-circATP9A group compared to that in the sh-NC group (Fig. 3A-C). As a result, nude mice injected with sh-circATP9A#1 exhibited smaller tumor volume at each time point than the sh-NC group (Fig. 3D). Subsequently, the expression levels of Ki67 were found to be consistent with circATP9A in mouse orthotopic tumor tissues by IHC (Fig. 3E). Meanwhile, the results of the lung metastasis model indicated that the lung tissue in the sh-circATP9A group has fewer lung metastases than the sh-NC group (Fig. 3F). Taken together, our data suggest that circATP9A facilitates the growth and metastasis of NSCLC in vivo.

### circATP9A directly binds to HuR

**Fig. 4:**
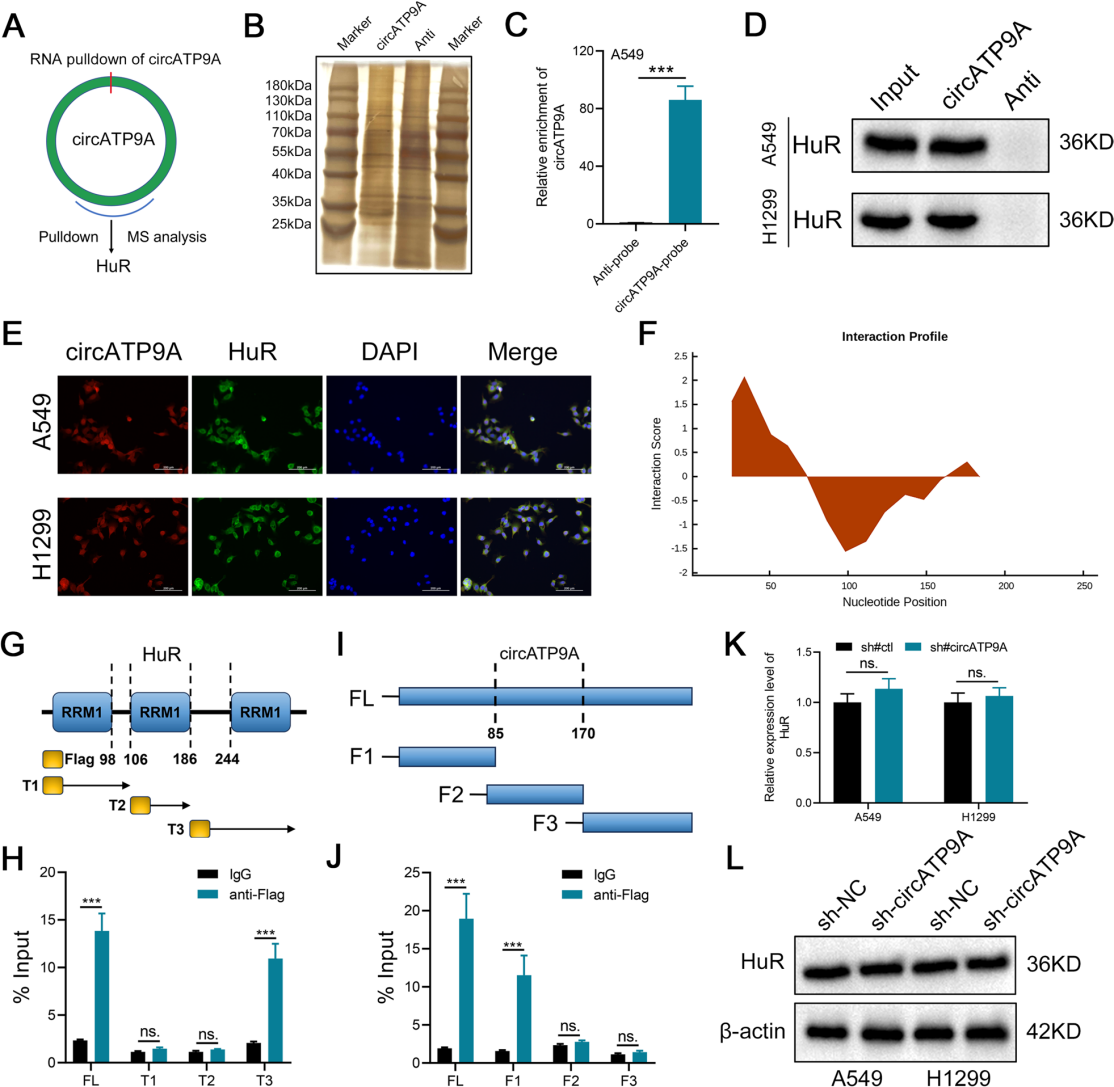
circATP9A direct interacts with HuR. Notes: **A** RNA pull-down assay was applied in NSCLC cells to identify the proteins that interacted with circATP9A;** B** The silver staining image of RNA pull-down with circATP9A probe in A549 cells; **C** qRT–PCR analysis confirmed that the circATP9A probe could specifically enrich circATP9A in A549 cells by RNA pull-down assay; **D** Western blot assay in circATP9A pull-down proteins confirmed the interaction between circATP9A2 and HuR; **E** The subcellular co-localization of circATP9A and HuR in NSCLC cells was measured by fluorescence staining assay; **F** The catRAPID was used to predict the binding site of circATP9A to HuR;** G-H** Schematic structures of HuR proteins and 3 truncations of HuR (top); RIP assays confirmed the interaction of truncation 3 of HuR with circATP9A in A549 cells (bottom);** I-J** Schematic structures of circATP9A and 3 fragments (top); RIP assays confirmed the interaction of fragment 1 of circATP9A with HuR in A549 cells (bottom); **K** The mRNA expression level of HuR in the circATP9A knockdown and control cells; **L** The protein expression level of HuR in the circATP9A knockdown and control cells

To explore the molecular mechanism by which circATP9A induced progression in NSCLC, a biotin-coupled circATP9A probe and control probe were used through RNA pull-down assay to identify the proteins interacting with circATP9A in A549 and H1299 cells (Fig. 4A). Silver staining results showed that an obvious band between 25 and 40 kDa was abundantly enriched in the circATP9A probe group (Fig. 4B). Additionally, circATP9A was validated as being specifically enriched in the circATP9A probe group through qRT–PCR assay (Fig. 4C). Then, MS analysis confirmed that HuR was enriched in the circATP9A probe group (Supplementary file 1). Western blot of the RNA pull-down proteins showed that circATP9A could specifically bind to HuR (Fig. 4D). Moreover, FISH-IF assay through confocal microscopy demonstrated that circATP9A and HuR were colocalized mostly in the cytoplasm of A549 and H1299 cells (Fig. 4E). Then catRAPID (http://service.tartaglialab.com/page/catrapid_group) was used to predict the interaction region between circATP9A and HuR, results showed that 26–85-nt region of circATP9A was required for HuR interaction (Fig. 4F and Figure S3). HuR is composed of three RNA recognition motifs (RRMs). To investigate its interactions, we generated three Flag-tagged vectors encoding overlapping truncations of HuR, particularly in its inactive sections. Subsequently, A549 cells were co-transfected with circATP9A and these vectors, with RIP assays demonstrating that circATP9A chiefly binds’o HuR's RRM1 region (Fig. 4G-H). We further engineered four vectors encoding fragments of circATP9A and co-transfected them with the Flag-tagged HuR plasmid into A549 cells. The RIP assay outcomes suggested that fragment 1 (F1) of circATP9A interacts with HuR, aligning with the predicted binding regions (Fig. 4I-J). Moreover, we found that knocking down circATP9A did not affect the protein and RNA level of HuR (Fig. 4K-L). Meanwhile, results from public data showed that HuR is overexpressed in most cancers (Figure S4A-B), and correlated with poor prognosis (Figure S4C-E).

### NUCKS1 is the target gene of circATP9A/HuR complex

**Fig. 5:**
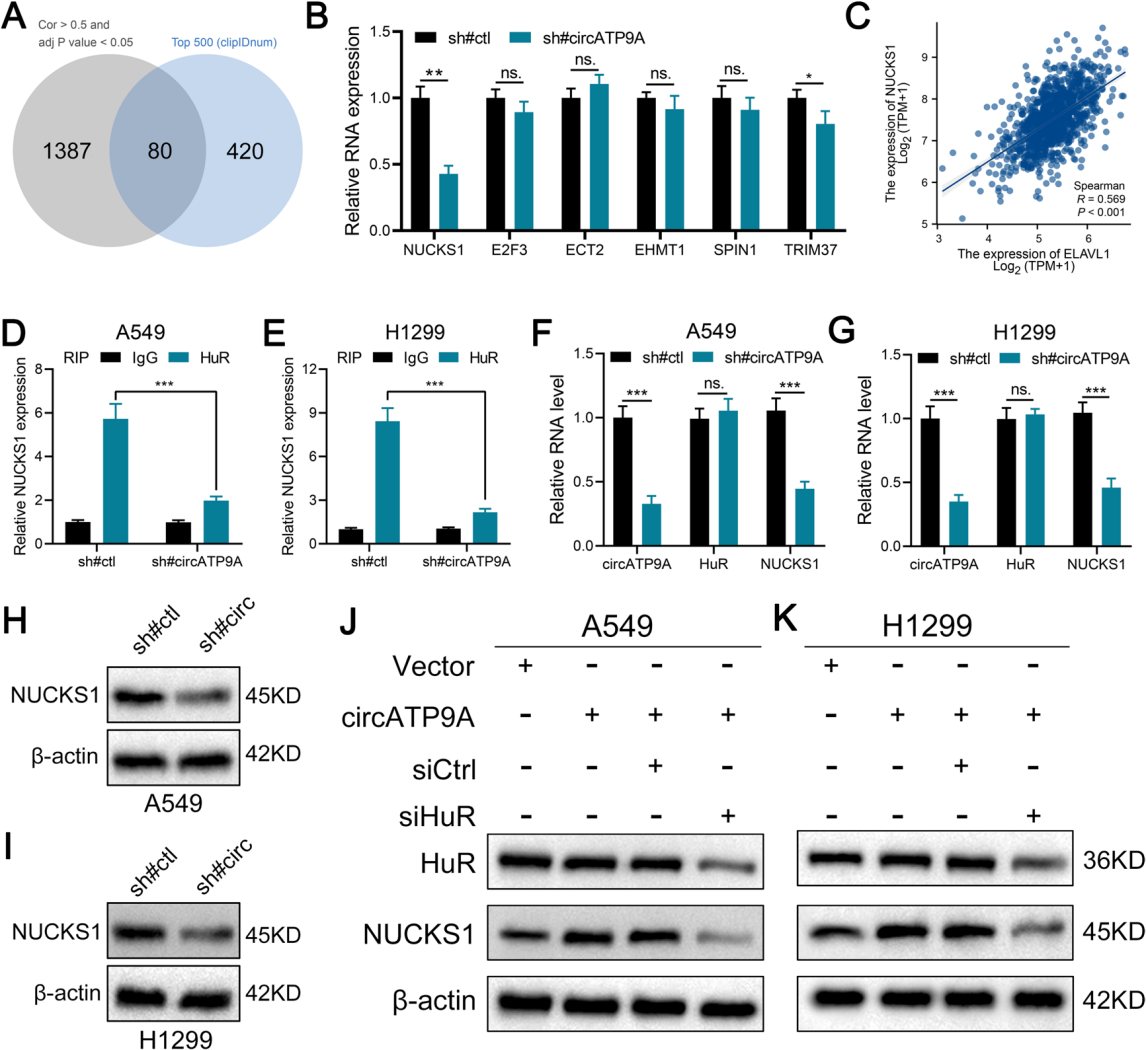
NUCKS1 is the target gene of circATP9A/HuR complex. Notes: **A** Intersection results of correlation analysis and clip-seq data identified 80 molecules; **B** Expression level of NUCKS1, E2F3, ECT2, EHMT1, SPIN1 and TRIM37 in the circATP9A knockdown and control cells; **C** Correlation between NUCKS1 and HuR based on TCGA database; **D-E** RIP assay was performed in A549 and H1299 cells to detect the NUCKS1 expression enriched by IgG and HuR; **F-G** The expression level of circATP9A, HuR and NUCKS1 in the circATP9A knockdown and control cells; **H-I** The protein level of NUCKS1 in the circATP9A knockdown and control cells; **J-K** Western blot assay was performed to measure HuR and NUCKS1 protein level after transfection of HuR siRNA, control siRNA in circATP9A overexpressed NSCLC cells

Previous studies have shown that HuR can directly bind with circRNA, further regulating the stability of downstream genes [18–20]. Our results have indicated that HuR could directly bind with the circATP9A. Therefore, we speculate that circATP9A can exert its cancer promoting effect by binding to HuR, affecting the RNA expression of downstream effector molecules. For this, we obtained the clip-seq data (HuR) and expression profile data from TCGA database. We found that a total of 1467 molecules have a strong positive correlation with HuR (Cor > 0.5 and *P* < 0.05) (Supplementary file 2). Then, we also extracted the top 500 genes with a large number of binding sites to HuR protein based on the clip-seq data (Supplementary file 3). We noticed that 80 genes were significantly positively correlated with HuR and also have a large number of binding sites to HuR protein (Fig. 5A). Among these genes, NUCKS1, E2F3, ECT2, EHMT1, SPIN1 and TRIM37 have been found to promote the progression of NSCLC, therefore they were included in our candidate gene list. Then, we detect the RNA level of above genes in the sh#ctl and sh#circATP9A cells. Results showed that NUCKS1 was remarkably downregulated in the cells with circATP9A knockdown (Fig. 5B). Moreover, NUCKS1 exhibit a significant positive correlation at the tissue RNA level (Fig. 5C, Cor = 0.569, *P* < 0.001). Then, we performed RIP assay to validate the interaction between NUCKS1 and HuR protein. Results showed that NUCKS1 can directly bind to HuR protein, yet the effect can be weakened by the knockdown of circATP9A (Fig. 5D-E). Meanwhile, we found the knockdown of circATP9A could significantly downregulate the RNA level of NUCKS1, but has no effect on circATP9A (Fig. 5F-G). Meanwhile, we noticed that the knockdown of circATP9A could also reduce the protein level of NUCKS1 (Fig. 5H-I). However, the positive effect of circATP9A on NUCKS1 protein can be inhibited with the HuR knockdown (Figure S5A and Fig. 5J-K). Moreover, we found that overexpressing circATP9A in NSCLC cells with HuR knockdown did not alter the protein expression of NUCKS1 compared to the Vector group (Figure S5B).

### circATP9A promotes NSCLC malignant phenotype by partly regulating NUCKS1 and PI3K/AKT/mTOR signaling

**Fig. 6:**
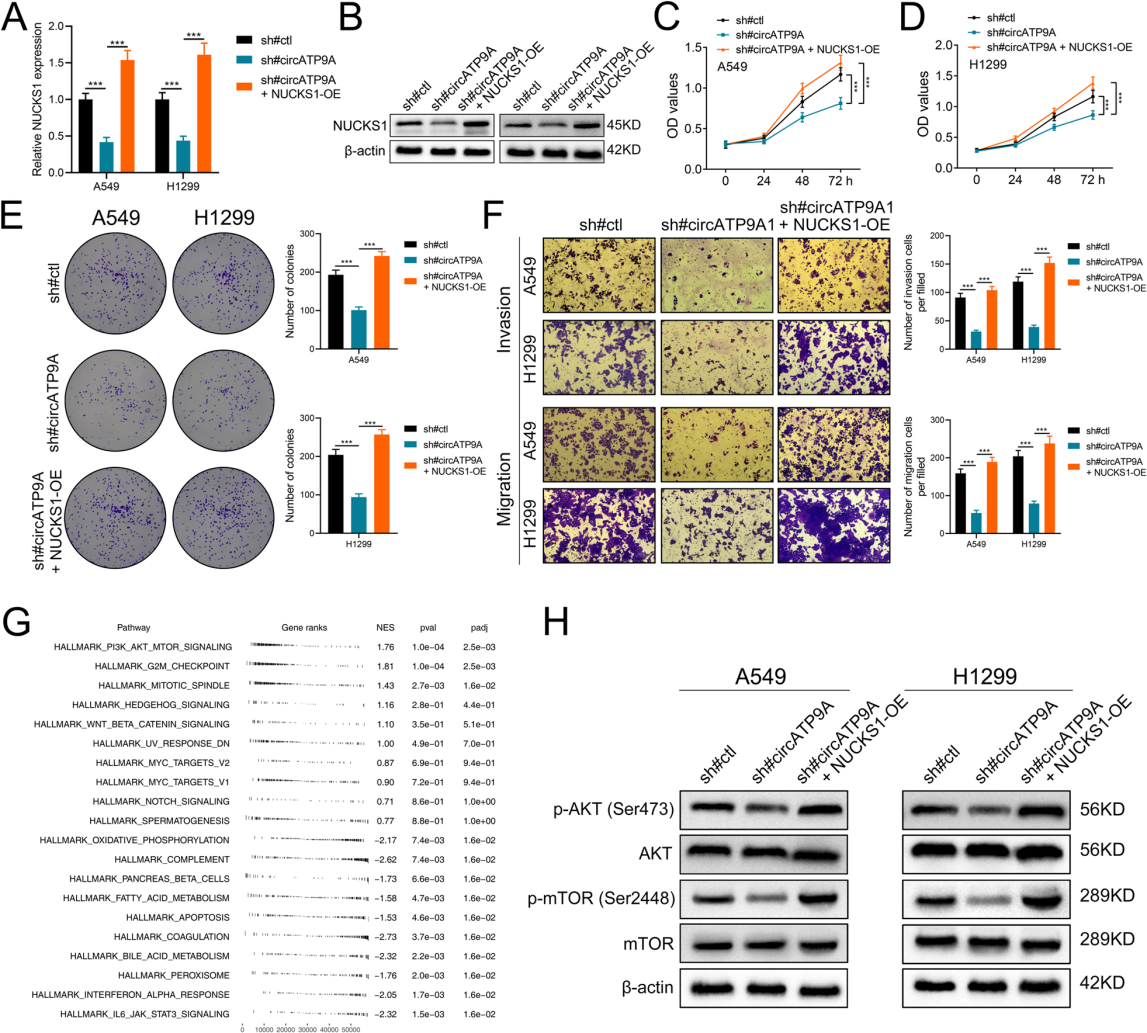
circATP9A governs NSCLC growth and metastasis by stabilizing NUCKS1 mRNA in vitro. Notes: **A**,** B** The NUCKS1 mRNA and protein level analyzed by qRT-PCR and western blot after re-express NUCKS1 in circATP9A knockdown NSCLC cells; **C**,** D** CCK8 assays for effects of NUCKS1 re-expression on proliferation of A549 and H1299 cells with circATP9A knockdown; **E** Colony formation assay for effects of NUCKS1 re-expression on proliferation of A549 and H1299 cells with circATP9A knockdown;** F** Transwell assay for effects of NUCKS1 re-expression on invasion and migration of A549 and H1299 cells with circATP9A knockdown; **G** fgsea analysis of NUCKS1 in NSCLC; **H** The protein level of the markers of PI3K/AKT/mTOR signaling in the A549 and H1299 cells with NUCKS1 re-expression with circATP9A knockdown

Our hypothesis was that NUCKS1 partly mediated the role of circATP9A in NSCLC. To verify this, we reintroduced NUCKS1 into NSCLC cells with circATP9A knockdown (Fig. 6A-B). The reintroduction of NUCKS1 significantly revived the proliferation capacities of both A549 and H1299 cells (Fig. 6C-E). Moreover, when we restored NUCKS1 expression in cells with circATP9A knockdown, there was a noticeable enhancement in the migratory and invasive potential of the cell lines under investigation (Fig. 6F). To identify the underlying downstream signaling of circATP9A/HuR/NUCKS1 axis, we conducted GSEA analysis based on Hallmark gene set. Results showed the the most enriched terms is PI3K/AKT/mTOR signaling in the patients with high NUCKS1 expression (Fig. 6G). As a classic cancer pathway, PI3K/AKT/mTOR signaling has been reported to be involved in the progression of NSCLC [21]. WB results showed that the inhibition of circATP9A can significantly suppress the activity of PI3K/AKT/mTOR signaling, yet re-expressed NUCKS1 can remarkably remedy this effect (Fig. 6H). Taken together, our results showed that circATP9A can directly bind with HuR to increase the expression level of NUCKS1, further activating PI3K/AKT/mTOR signaling and promote cancer progression.

### hnRNPA2B1 regulated circATP9A packaging into EVs

**Fig. 7:**
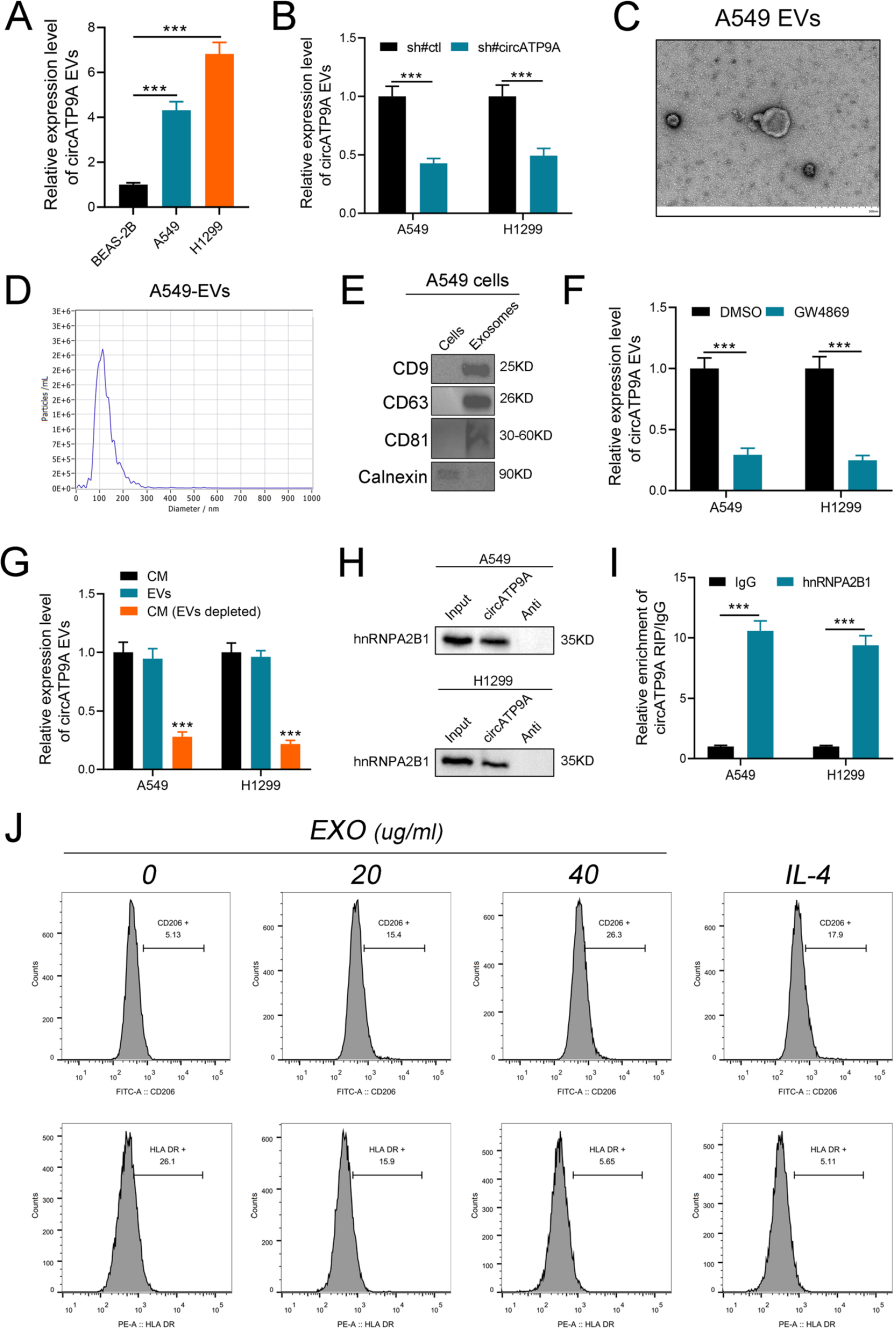
hnRNPA2B1 mediates the packaging of circATP9a2 into EVs. Notes:** A** qRT-PCR analysis of the expression level of EVs-circATP9A in BEAS-2B, A549, and H1299 cells; **B** The expression level of EVs-circATP9A in NSCLC cells after knockdown of circATP9A; **C**,** D** Transmission electron microscopy (TEM) and NanoSight were used to characterize the purified EVs from A549 cells; **E** Western blot analysis of EVs markers from A549 EVs or cell lysates; **F** qRT-PCR analysis of the expression level of circAtP9A in EVs from NSCLC cells treatment with GW4869 (an inhibitor of EVs secretion); **G** qRT-PCR analysis of the expression level of circATP9A in the CM of NSCLC cells after depletion of EVs by ultracentrifugation; **H** Western blot assay confirming the interaction between circATP9A and hnRNPA2B1 in circATP9A pull-down proteins;** I** RIP assay in A549 cells confirmed that circATP9A could be enriched by hnRNPA2B1; **J** Flow cytometric analysis of the expressions of CD206/HLA-DR in macrophages treated with different concentrations of exosomes isolated from supernatants of NSCLC cells. Numerical values denote the relative fluorescence intensity

Previous studies showed that EVs were involved in the progression of various tumors [22]. Interestingly, we found increased EVs-circATP9A in NSCLC cell lines compared to BEAS-2B cells (Fig. 7A). Moreover, the enrichment of circATP9a in EVs was closely relative to its expression in NSCLC cells, indicating that EVs-circATP9A may play a vital role in the metastasis of RCC (Fig. 7B-C). In addition, classic characteristics of EVs were observed in the EVs derived from A549 and H1299 cell lines: a typical cup-shaped morphology, approximately 30–150 nm in size, and typical protein markers CD9, CD63, CD81, and Calnexin (Fig. 7C-E). To verify the existence of extracellular circATP9A mainly in the form of EVs, GW4869 was used to inhibit EVs secretion. The results showed that the level of EVs-circATP9A was significantly downregulated after treatment with GW4869, while no effects on the level of circATP9A in NSCLC cells (Fig. 7F). Notably, qRT-PCR was performed to detect the level of circATP9A in the culture medium, EVs, and EVs-depleted culture medium (purified by ultracentrifugation). Results showed that the level of circATP9A was significantly lower in the EVs-depleted culture medium than in EVs and total culture medium (Fig. 7G). Taken together, these results indicate that the extracellular form of circATP9A mainly exists in EVs. We then explore the mechanism by which circATP9A was selectively packaged into EVs. MS analysis confirmed that hnRNPA2B1 was enriched in the circATP9A probe group (Supplementary file 1). Then, western blot of RNA pull-down proteins and RIP assay also confirmed the interaction between circATP9A and hnRNPA2B1 (Fig. 7H-I). As previously reported, hnRNPA2B1 could regulate the packaging of RNAs into EVs by recognizing the specific motifs GGAG/CCCU [23]. In particular, we recognized the GGAG motif in the sequence of circATP9A. Moreover, we found that EVs-circATP9A was downregulated after the knockdown of hnRNPA2B1, while the level of circATP9A in NSCLC cells was not significantly affected (Figure S5C-D). These results revealed that circATP9A could package into EVs in an hnRNPA2B1-dependent manner.

### NSCLC cell-derived EVs regulate macrophage M2 polarization

**Fig. 8:**
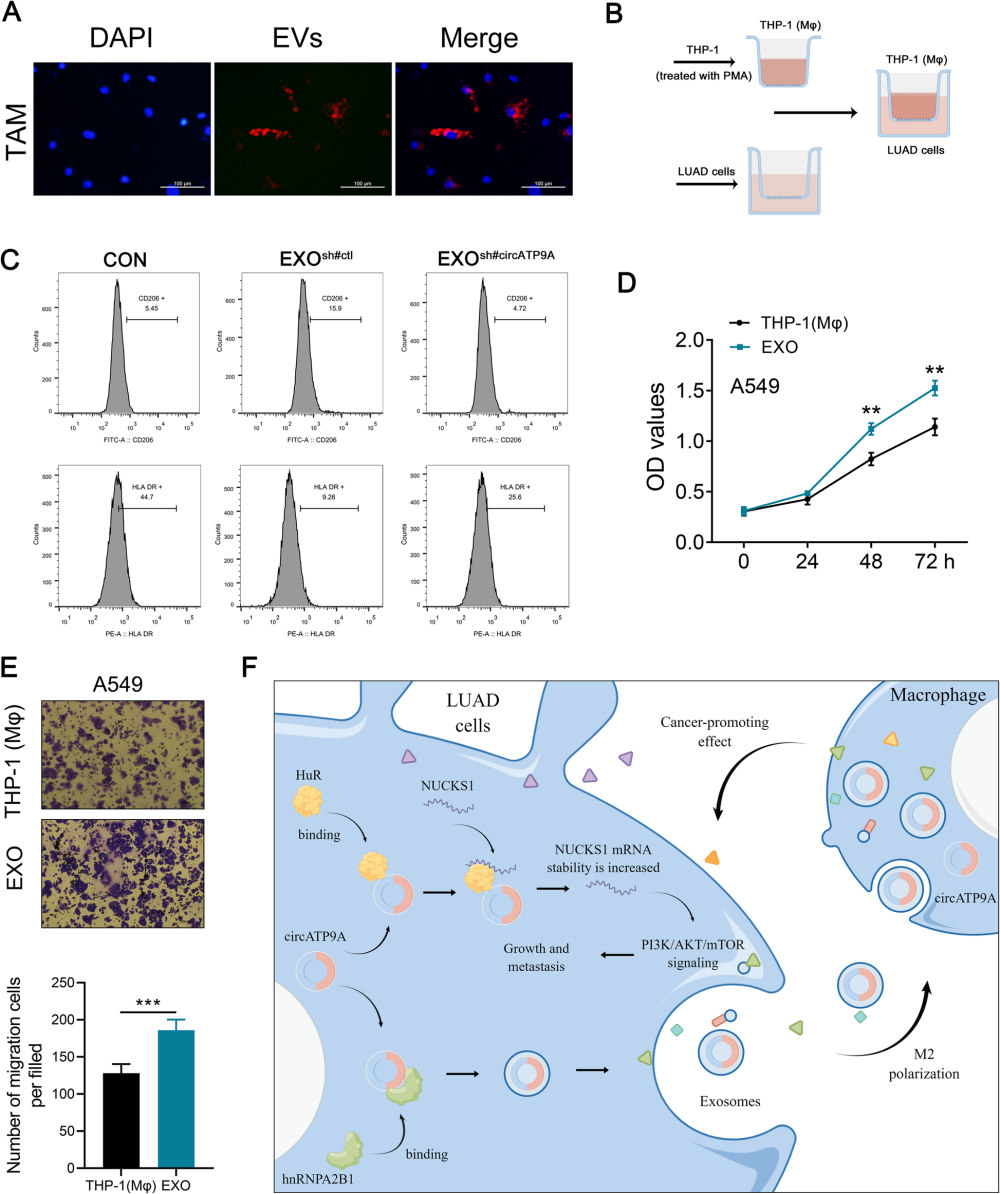
NSCLC cell-derived exosomal circATP9A induces macrophages M2 polarization. Notes: **A** Representative images of A549 cells after incubation with PKH26-labeled NSCLC-EVs; **B** Co-cultivation mode diagram; **C** Flow cytometric analysis of the expressions of CD206/HLA-DR in macrophages treated with exosomes with different circATP9A levels. Numerical values denote the relative fluorescence intensity; **D** The proliferation ability of A549 cells was assessed by CCK8 assay [(EXO and co-cultured with T)P-1 (Mφ)]; **E** The invasion ability of A549 cells was assessed by CCK8 assay [(EXO and co-cultured with T)P-1 (Mφ)]; **F** A schematic model of this study

Exosomes are crucial to cellular interaction as they transport circRNAs, and TAMs are the predominant cell type in the tumor microenvironment [24]. As such, we speculated that exosomal circATP9A plays a significant role in mediating the dialogue between cancer cells and TAMs [25]. To initiate our functional assays, THP-1 (Mφ) cells were exposed to either 20 or 40 μg/ml of exosomes extracted from A549 cellls (with 20 ng/ml IL-4 treatment serving as a positive control) for 24 h. Upon exosome exposure, these cells displayed a CD206 high/HLA-DRlow– M2 phenotype (Fig. 7J), suggesting an activated state. Then, EVs were purified from A549 cells and labeled with PKH26 (red), then cocultured with THP-1 (Mφ) cells for 24 h. After incubation, the red fluorescence signal was observed in the cytoplasm of recipient THP-1 cells, indicating that EVs secreted by NSCLC cells could deliver to THP-1 (Mφ) cells (Fig. 8A). To investigate whether NSCLC cells derived from EVs-circATP9A could promote macrophage M2 polarization, a coculture system with THP-1 (Mφ) cells was established (Fig. 8B). As anticipated, compared to the circATP9A knockdown control and blank control groups, exosomes derived from A549 cells with circATP9A knockdown were observed to increase the expression of M1 markers and decrease M2 markers. (Fig. 8C). Then, we performed functional assays in the A549 cells co-cultured with THP-1 (Mφ) cells with nothing added or THP-1 (Mφ) with 40 μg/ml exosomes added. CCK8 and transwell assays both indicated that the A549 cells co-cultured with exosomes showed more progressive biological behavior (Fig. 8D-E). We initiated lung cancer in immunocompetent C57/B6 mice using a tail vein injection method. Subsequently, we administered exosomes derived from both control and A549 cells with circATP9A overexpressing, according to a pre-established protocol. Immunofluorescence analysis revealed a marked increase in the expression of the M2 macrophage marker CD206 in mice that received exosomes from the circATP9A-overexpressing A549 cells (Figure S6A). This finding was corroborated by flow cytometry (Figure S6B), further confirming that exosomes carrying circATP9A can effectively induce M2 macrophage polarization within the lung cancer microenvironment in vivo.

## Discussion

Circular RNAs (circRNAs) have garnered substantial interest due to their unique stability and diverse expression profiles, marking them as potential contributors in the field of cancer research [26]. As potential oncological markers, circRNAs can be utilized for the early identification of cancer, the evaluation of prognosis, and the monitoring of treatment responses [27]. Given that circRNA expressions within cells are often tissue-specific, it affords the opportunity for precise diagnoses of various cancer types [28]. Concurrently, certain circRNAs have been found to influence the growth, migration, and invasion of cancer cells, suggesting the possibility of leveraging circRNAs as novel therapeutic targets [29]. Nevertheless, despite the promising potential of circRNAs as cancer markers, further research is imperative to substantiate and deepen our understanding of their specific roles within oncology [30]. In the present study, we noticed a considerable amplification of circATP9A in NSCLC tissue samples, particularly in those at more advanced clinical stages. These findings strongly propose the potential role of circATP9A as a meaningful tissue biomarker in NSCLC progression. However, the study is constrained by the limited number of patients involved and the relatively brief inclusion period. We aim to increase patient enrollment and continue patient follow-ups to acquire survival data, thereby evaluating the efficacy of circATP9A as a prognostic indicator in NSCLC. Further, we could probe the expression levels of circATP9A in serum in future studies to ascertain its potential as a liquid biopsy biomarker.

Our study elucidates the functional role of circATP9A in NSCLC. We demonstrated that circATP9A can foster the progression of NSCLC. From a mechanistic standpoint, circATP9A can interact with the HuR protein to form an RNA–protein complex, subsequently amplifying the mRNA and protein levels of the target gene NUCKS1. Further, the PI3K/AKT/mTOR signaling was identified as the downstream pathways of circATP9A/HuR/NUCKS1 axis. More notably, hnRNPA2B1 can mediate the incorporation of circATP9A into EVs. Subsequently, these EVs containing circATP9A induce the M2 phenotype of TAMs, thereby facilitating NSCLC development (Fig. 8F).

Within cells, circRNAs can interact with RNA-binding proteins (RBPs) [31]. RBPs are a category of proteins that bind to RNA molecules, governing RNA metabolism and functionality [32]. The association of circRNA with RBP can influen’e RBP's functionality and the stability of RNA [33]. The complexity and intrigue of circRNA-protein interaction exceed that of circRNA-miRNA interaction, attracting recent research focus [34]. RBPs exert influence over all phases of the circRNA life cycle, including its biogenesis, localization, functionality, and degradation.

HuR, a key RNA-binding protein, forms complexes with a wide variety of RNA molecules, including mRNA, non-coding RNA, and others, subsequently influencing their stability, localization, and translation [35]. By recognizing and binding to specific sequences of RNA (usually AU-rich regions), HuR significantly determines the fate of RNA [36]. In the context of cancer, overexpression or malfunctions of HuR frequently give rise to malignant phenotypes, including amplified cell proliferation, apoptosis resistance, and enhanced migratory ability [37, 38]. Furthermore, HuR partakes in numerous physiological and pathological processes such as immune response and stress response, positioning itself as a pivotal regulator of RNA metabolism and messenger RNA [39]. In our research, we identified the interaction between circATP9A and HuR via bioinformatic analysis and substantiated this interplay through subsequent pulldown assays. Moreover, we found that escalated circATP9A expression encourages NSCLC progression through its engagement with HuR, thus amplifying downstream NUCKS1 expression. Yet, the precise mechanistic details warrant further investigation. Concurrently, we cannot disregard other potential mechanisms such as those involving miRNA sponges.

Recently, more and more studies have shown that circRNAs can interact with regulatory RBPs and further affect the fate of their target mRNAs [40]. Our results suggest that NUCKS1 is regulated by the circATP9A/HuR complex. NUCKS1 is a non-histone chromatin structural protein widely present in a variety of organisms [41]. NUCKS1 plays an important role in cell proliferation, DNA damage repair and signal transduction. Studies in recent years have shown that NUCKS1 is upregulated in some types of cancer and may be related to tumorigenesis and progression, so it is considered a potential tumor biomarker and therapeutic target [42]. In NSCLC, Zhao et al. noticed that NUCKS1 can promote NSCLC proliferation, invasion and migration through upregulating CDK1 expression [43]. Yu et al. demonstrated that circRNA circ_0008037 promotes tumor growth and the Warburg effect by upregulating NUCKS1 through binding to miR-433-3p in NSCLC [44]. Our investigation demonstrates that circATP9A associates with HuR, thereby enhancing its expression, which in turn results in increased regulation of NUCKS1 mRNA. However, our current experimental data indeed demonstrate an interaction between RNA and protein, but they do not conclusively establish whether this interaction is direct or mediated by other molecules. Meanwhile, it is important to note that the influence of circATP9A on NSCLC cells cannot be entirely attributed to NUCKS1 alone, suggesting the existence of other mechanisms. Our results demonstrate that circATP9A directly binds with HuR to increase NUCKS1 expression, subsequently activating PI3K/AKT/mTOR signaling and promoting cancer progression. Nevertheless, the incomplete reversal of NSCLC malignancy phenotypes by NUCKS1 reintroduction implies additional circATP9A-related pathways or mechanisms that require further exploration.

According to previous studies, non-cancerous cells present within the tumor microenvironment can significantly contribute to tumor progression [45]. Notably, benign macrophages situated in the tumor microenvironment have been identified to exacerbate malignant progression, facilitating angiogenesis, invasiveness, and migration of cancer cells while hampering antitumor immunity [46]. Macrophages, being critical immune cells in the tumor milieu, are often polarized into two distinct phenotypes: M1 or M2. These variants execute different roles, with M1 macrophages generally exerting tumor-suppressive functions, while M2 macrophages encourage tumor development. Importantly, Guo et al. noticed that the M2 phenotype induced by THP-1 cells can promote NSCLC cell metastasis [47].

EVs are a diverse group of minute vesicles, with sizes spanning from 30 to 1000 nm [48]. They are secreted by cells and are packed with biomolecules including proteins, lipids, RNA, and DNA [49]. In the context of cancer, EVs hold substantial significance. Cancer cells can secrete EVs that ferry these biomolecules to designated cells, thereby altering the biological activities of these recipient cells. For instance, EVs released by cancer cells can transport growth and metastasis-promoting signaling molecules, thereby influencing the conduct of both neighboring normal cells and distant recipient cells. Moreover, EVs may impact the tumor microenvironment, such as by inhibiting immune responses and boosting angiogenesis [50]. Therefore, EVs play a pivotal role in the onset and development of cancer. In our research, we discovered that circATP9A can be encapsulated into EVs, subsequently being engulfed by TAMs [51]. This process fosters their polarization towards an M2 phenotype, further expanding the functional spectrum of circATP9A. Nonetheless, the precise mechanism underlying this polarization warrants further investigation in subsequent studies.

The RNA-binding protein hnRNPA2B1 has been identified to play a role in the encapsulation of RNA into EVs by recognizing specific motifs, such as GGAG/CCCU [52]. Herein, we identified the presence of the GGAG motif within the sequence of circATP9A. Additionally, we observed a decreased expression of circATP9a in EVs after the knockdown of hnRNPA2B1. These results indicated that EVs-circATP9A may serve as a distinctive tactic for the treatment of NSCLC.

In conclusion, Our study's elucidation of the functional role of circATP9A in NSCLC presents several significant implications for the treatment and management of this disease. By demonstrating that circATP9A fosters the progression of NSCLC through its interaction with the HuR protein and the subsequent amplification of NUCKS1 mRNA and protein levels, we have uncovered a novel regulatory mechanism in lung cancer pathology. The identification of the PI3K/AKT/mTOR signaling pathway as a downstream target of the circATP9A/HuR/NUCKS1 axis further expands our understanding of the molecular underpinnings of NSCLC. From a therapeutic standpoint, these insights provide a foundation for developing new strategies targeting circATP9A. Inhibiting the function or expression of circATP9A could potentially disrupt the circATP9A/HuR/NUCKS1 signaling axis, thereby impeding NSCLC progression. This approach could be particularly effective in tumors where this pathway is upregulated. Moreover, our findings about the role of hnRNPA2B1 in mediating the incorporation of circATP9A into EVs and its subsequent influence on TAMs open up new avenues for therapy. Targeting these EVs or modulating the M2 polarization of TAMs could provide an innovative approach to altering the tumor microenvironment in favor of therapeutic efficacy. The potential of circATP9A as a diagnostic biomarker also cannot be overlooked. Its presence and levels in patient samples could serve as an indicator of disease progression or response to therapy, aiding in personalized treatment planning. In summary, our discoveries highlight circATP9A as a promising candidate for both diagnostic and therapeutic applications in NSCLC. Targeting this molecule could lead to the development of new therapeutic strategies that improve treatment outcomes for patients suffering from this challenging disease.

### Supplementary Information

**Additional file 1. **

**Additional file 2. **

**Additional file 3. **

**Additional file 4: Figure S1. **The expression level of circATP9A and linear ATP9A in cells with different treatment.

**Additional file 5: Figure S2. **Upregulation of circATP9A enhances the proliferation, invasion, and migration of NSCLC cells.

**Additional file 6: Figure S3. **The catRAPID was used to predict the binding site of circATP9A to HuR. 

**Additional file 7: Figure S4. **The expression level and clinical role of HuR in the TCGA database.

**Additional file 8: Figure S5. **Expression level of HuR, hnRNPA2B1 and circATP9A EVs in cells with different treatment.

**Additional file 9: Figure S6. **Cancer-secreted circATP9A induces macrophage M2 polarization  in vivo.

**Additional file 10: Table S1. **The sh-RNAs, si-RNAs used in this study.

**Additional file 11: Table S2. **Primes used in qRT-PCR and PCR analysis in this study.

**Additional file 12: Table S3. **Probes of FISH, RNA pull-down and ISH in this study.

## Data Availability

The data used to support the findings of this study are available from the corresponding author upon request.

## Abbreviations

EVs: Extracellular vesicles
NSCLC: Non-small cell lung cancer
TAM: Tumor-associated macrophage
EGFR: Epidermal growth factor receptor
circRNAs: Circular RNAs
RBPs: RNA-binding proteins
