# Correction: circular RNA circATP9A promotes non-small cell Lung cancer progression by interacting with HuR and by promoting extracellular vesicles-mediated macrophage M2 polarization

**DOI:** 10.1186/s13046-023-02924-6

**Published:** 2023-12-19

**Authors:** Yuanshan Yao, Chunji Chen, Jing Wang, Haojie Xuan, Xiuxiu Chen, Zheng Li, Fuzhi Yang, Bin Wang, Siyun Lin, Saitian Li, Dongfang Tang, Libao Gong, Wen Gao

**Affiliations:** 1 Department of Thoracic Surgery, Shanghai Key Laboratory of Clinical Geriatric Medicine, HuaDong Hospital Affiliated to Fudan University, Shanghai, 200041 China; 2 Department of Abdominal Oncology, The Cancer Center of the Fifth Afliated Hospital, Sun Yat-Sen University, Zhuhai, Guangdong Province 519000 China

**Correction:** ***J Exp Clin Cancer Res*** **42, 330 (2023)**

10.1186/s13046-023-02916-6

Following publication of the original article [1], authors noticed and error in Fig. 8. The figure was not captured and correct figure is given below:

**Fig. 8:**
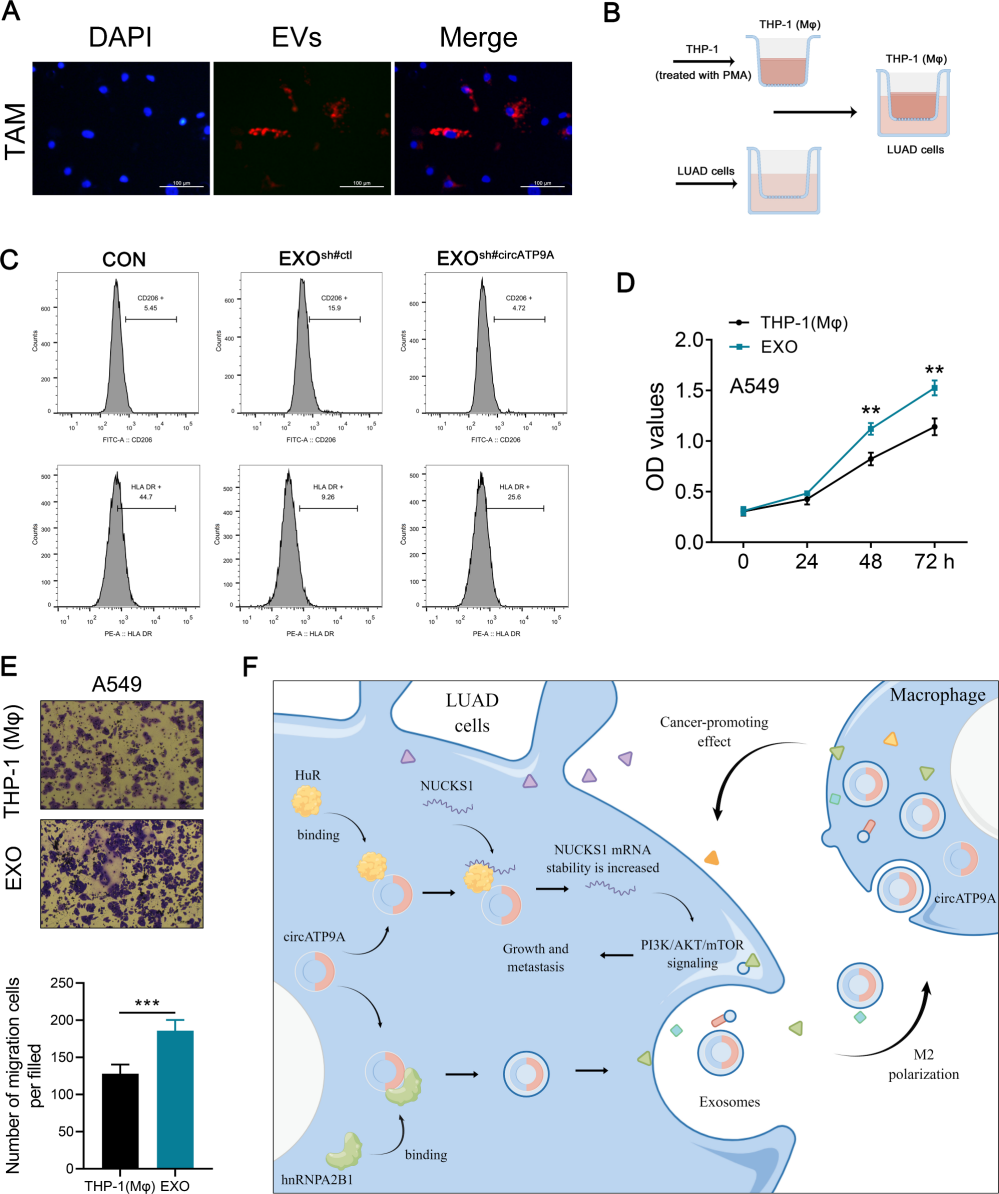
NSCLC cell-derived exosomal circATP9A induces macrophages M2 polarization. Notes: (**A**) Representative images of A549 cells after incubation with PKH26-labeled NSCLC-EVs; (**B**) Co-cultivation mode diagram; (**C**) Flow cytometric analysis of the expressions of CD206/ HLA-DR in macrophages treated with exosomes with different circATP9A levels. Numerical values denote the relative fluorescence intensity; (**D**) The proliferation ability of A549 cells was assessed by CCK8 assay [(EXO and co-cultured with T)P-1 (Mφ)]; (**E**) The invasion ability of A549 cells was assessed by CCK8 assay [(EXO and co-cultured with T)P-1 (Mφ)]; (**F**) A schematic model of this study

The correction does not affect the overall result or conclusion of the article. The original article has been corrected.
