# Supplementary figures and images for: Circular RNA circATP9A promotes non-small cell lung cancer progression by interacting with HuR and by promoting extracellular vesicles-mediated macrophage M2 polarization
Source: J Exp Clin Cancer Res. 2023 Dec 5;42:330. doi: 10.1186/s13046-023-02916-6 (PMC10696866; doi:10.1186/s13046-023-02916-6)

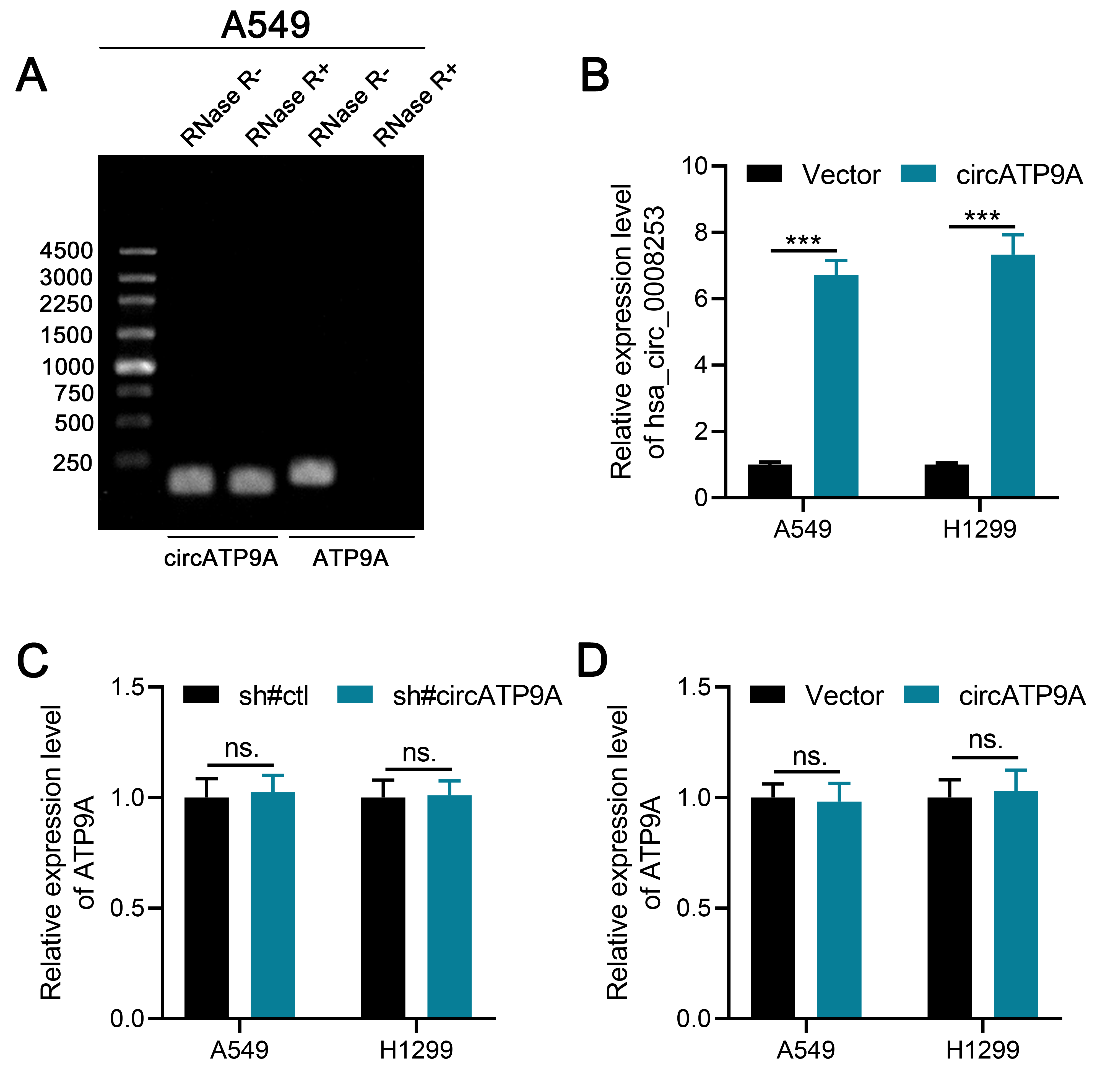

Supplement: Supplementary file 4 — Additional file 4: Figure S1. The expression level of circATP9A and linear ATP9A in cells with different treatment. [file 13046_2023_2916_MOESM4_ESM.tif]

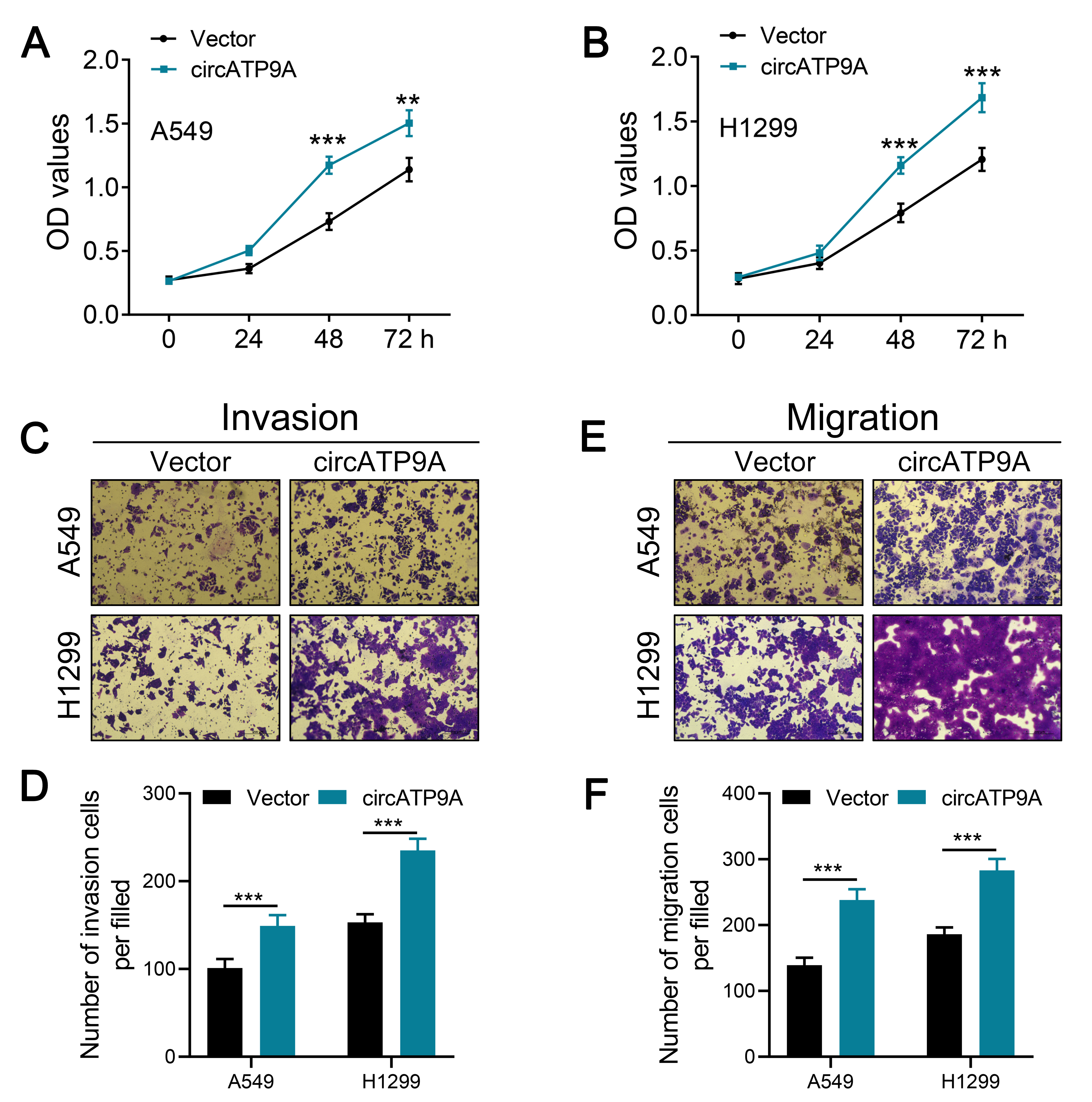

Supplement: Supplementary file 5 — Additional file 5: Figure S2. Upregulation of circATP9A enhances the proliferation, invasion, and migration of NSCLC cells. [file 13046_2023_2916_MOESM5_ESM.tif]

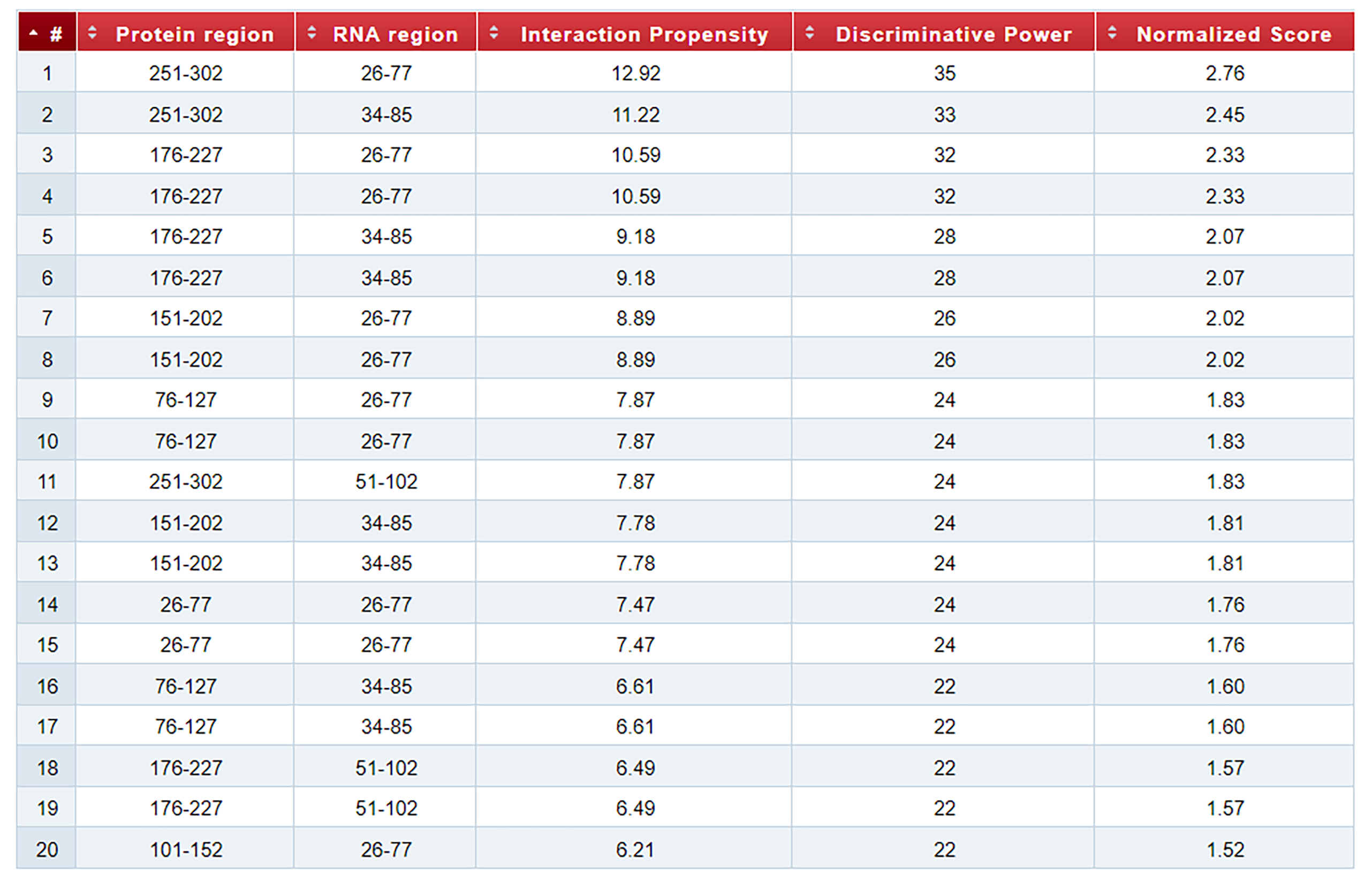

Supplement: Supplementary file 6 — Additional file 6: Figure S3. The catRAPID was used to predict the binding site of circATP9A to HuR. [file 13046_2023_2916_MOESM6_ESM.tif]

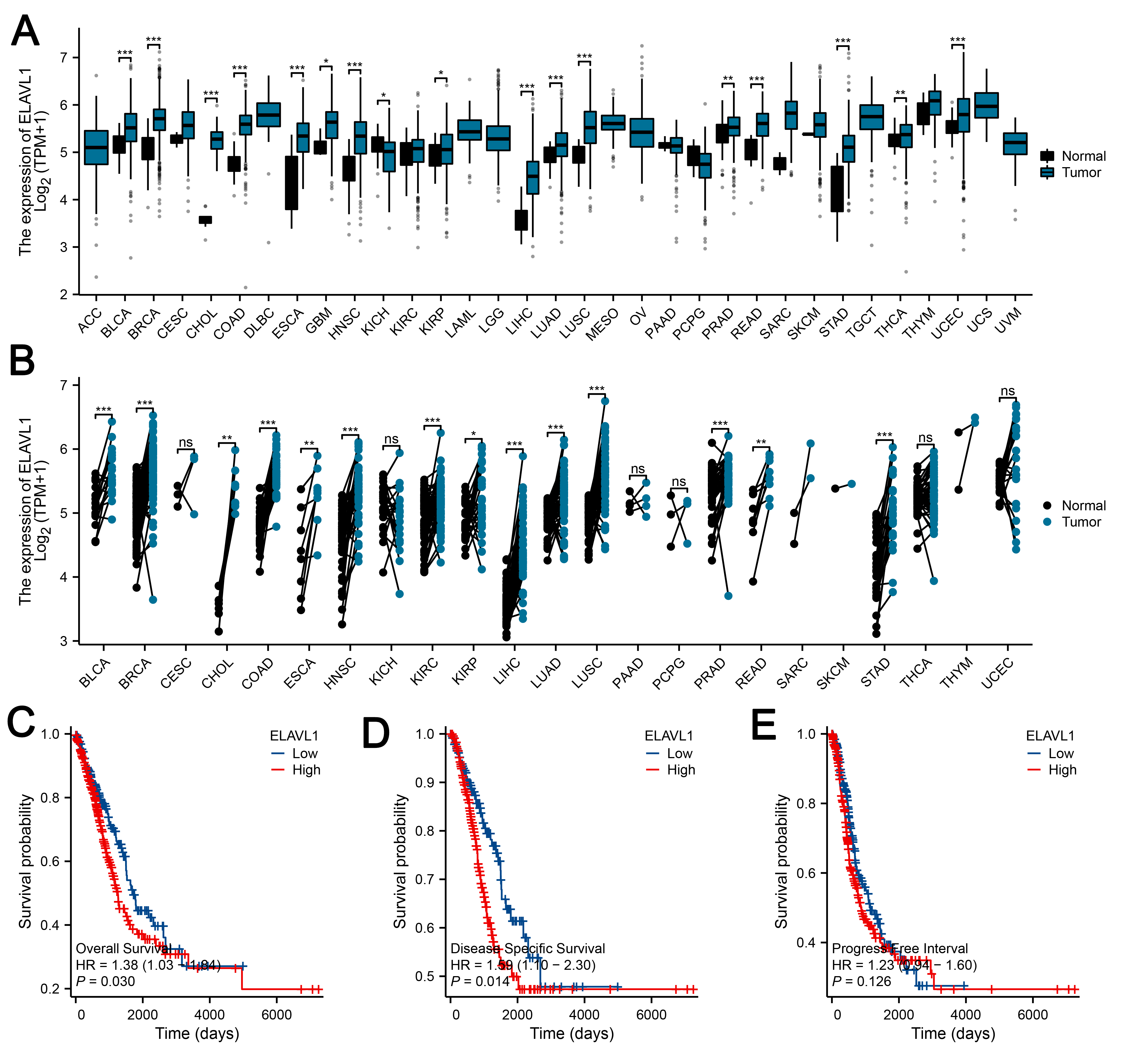

Supplement: Supplementary file 7 — Additional file 7: Figure S4. The expression level and clinical role of HuR in the TCGA database. [file 13046_2023_2916_MOESM7_ESM.tif]

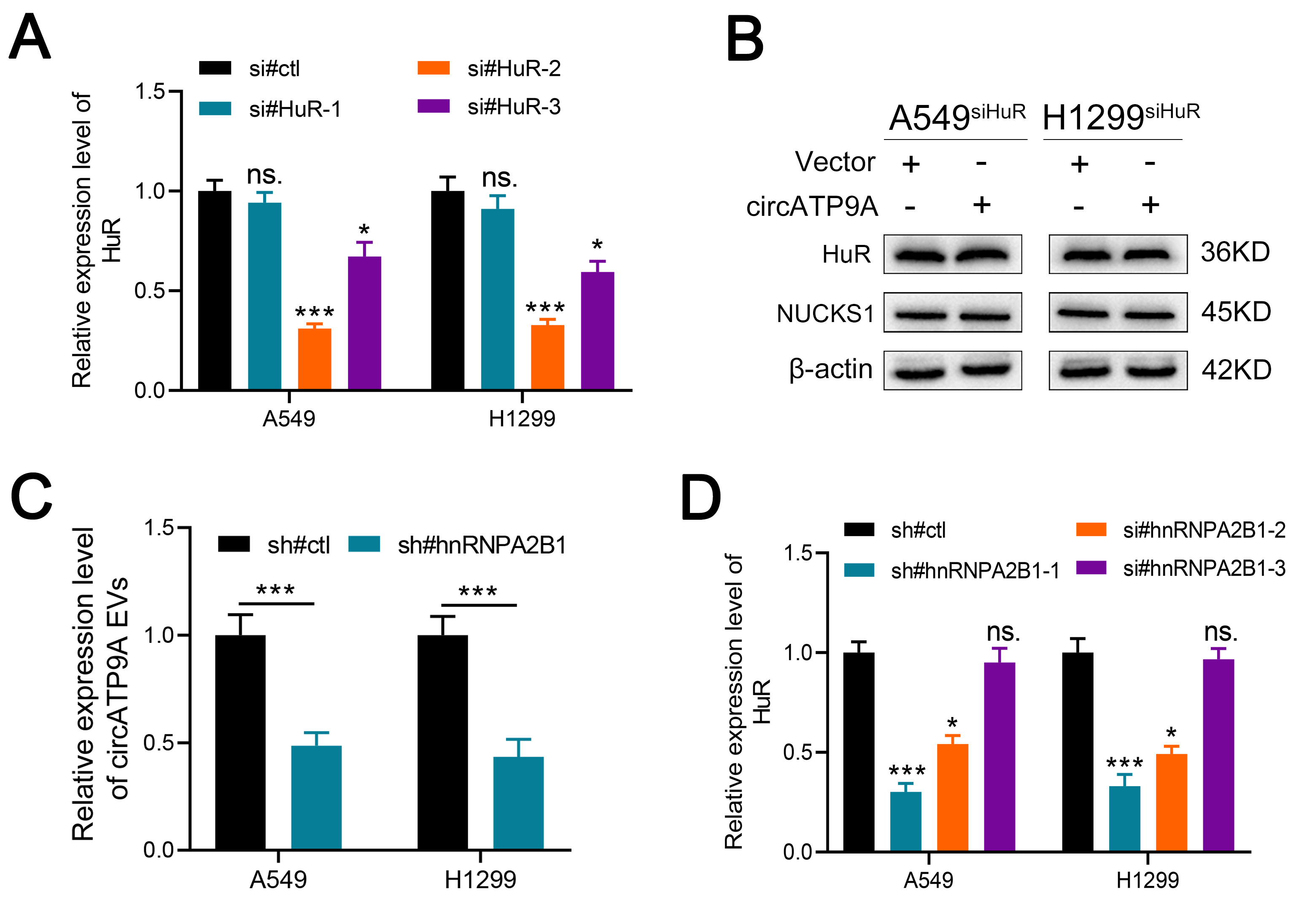

Supplement: Supplementary file 8 — Additional file 8: Figure S5. Expression level of HuR, hnRNPA2B1 and circATP9A EVs in cells with different treatment. [file 13046_2023_2916_MOESM8_ESM.tif]

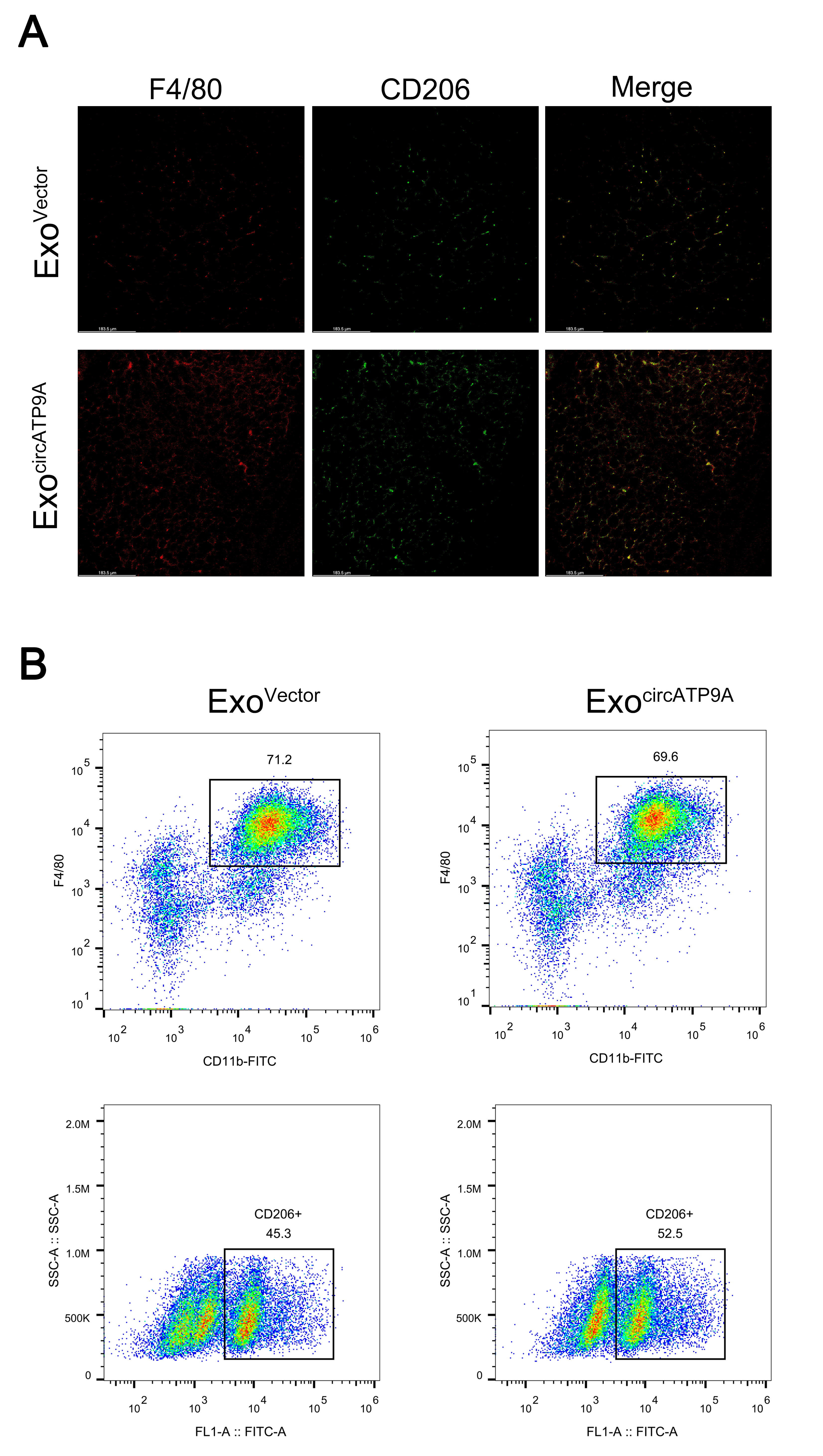

Supplement: Supplementary file 9 — Additional file 9: Figure S6. Cancer-secreted circATP9A induces macrophage M2 polarization in vivo. [file 13046_2023_2916_MOESM9_ESM.tif]
