# Supplementary material for: Circular RNA circATP9A promotes non-small cell lung cancer progression by interacting with HuR and by promoting extracellular vesicles-mediated macrophage M2 polarization
Source: J Exp Clin Cancer Res. 2023 Dec 5;42:330. doi: 10.1186/s13046-023-02916-6 (PMC10696866; doi:10.1186/s13046-023-02916-6)
Supplement: Supplementary file 10 — Additional file 10: Table S1. The sh-RNAs, si-RNAs used in this study. [file 13046_2023_2916_MOESM10_ESM.docx]

| Table S1. The sh-RNAs, si-RNAs used in this study | |
| --- | --- |
| sh-RNAs or si-RNAs | Sequence (5’-3’) |
| sh-NC | TTCTCCGAACGTGTCACGT |
| sh-circATP9A#1 | TTCCCCTGGTGCTGCGAGT |
| sh-circATP9A#2 | ACTGGGTTCCCCTGGTGCT |
| sh-circATP9A#3 | GGTTCCCCTGGTGCTGCGA |
| si-HuR#1 | CAGGGAAACTCCAGTATAT |
| si-HuR#2 | GCCCATAGCTAGCATTCAA |
| si-HuR#3 | GTGGAAGGATTTAGCTTAA |
| sh-hnRNPA2B1#1 | GGCGGAATTAAAGAAGATA |
| sh-hnRNPA2B1#2 | GGTGGCTTAAGCTTTGAAA |
| sh-hnRNPA2B1#3 | GGCTGCAAGACCTCATTCA |
