# Supplementary material for: Circular RNA circATP9A promotes non-small cell lung cancer progression by interacting with HuR and by promoting extracellular vesicles-mediated macrophage M2 polarization
Source: J Exp Clin Cancer Res. 2023 Dec 5;42:330. doi: 10.1186/s13046-023-02916-6 (PMC10696866; doi:10.1186/s13046-023-02916-6)
Supplement: Supplementary file 11 — Additional file 11: Table S2. Primes used in qRT-PCR and PCR analysis in this study. [file 13046_2023_2916_MOESM11_ESM.docx]

Table S2 Primes used in qRT-PCR and PCR analysis in this study

| Primers | Sequence (5’-3’) |
| --- | --- |
| GAPDH | F: CAGGAGGCATTGCTGATGAT |
|  | R: GAAGGCTGGGGCTCATTT |
| circATP9A | F: GACCAGAGGTATCCTCGGAATGTC |
|  | R: CCACTCGCAGCACCAGGG |
| ATP9A mRNA | F: CTCTGCCTACCACCATTCCA |
|  | R: GGGCTGTAGCACCAAGAACC |
| circATP9A (divergent) | F: TGCCTGCTCTCAGTTTGTTCC |
|  | R: ATACCTCTGGTCTCTCTTCTCGG |
| circATP9A (convergent) | F: CACCCCGAGAAGAGAGACCA |
|  | R: TTCGGGAACAAACTGAGAGCA |
| HuR | F: TGGGCATTGCTGGACTGAT |
|  | R: CTTTCGCCTAGATTTCCCATAGAT |
| hnRNPA2B1 | F: CAACCTTCTAACTACGGTCCAA |
|  | R: CAGTATCGGCTCCTCCCAC |
| NUCKS1 | F: GGCCTGTCAGAAATAGGAAGGT |
|  | R: TTTAGCTTCTCGGGGAGATGAT |
| E2F3 | F: AGAAAGCGGTCATCAGTACCT |
|  | R: TGGACTTCGTAGTGCAGCTCT |
| ECT2 | F: ACTACTGGGAGGACTAGCTTG |
|  | R: CACTCTTGTTTCAATCTGAGGCA |
| EHMT1 | F: GCTGTGTGAAAACCGAGCTG |
|  | R: TCCGCTATCCGAGTTAGTGTG |
| SPIN1 | F: CAGTGTGGGTCCGAGCAAA |
|  | R: CAGGGCCATTCCCCTCTTT |
| TRIM37 | F: TATGGAGAAATTGCGGGATGC |
|  | R: GTCAGCCAGCGCCTAATACAG |
