# Supplementary material for: Circular RNA circATP9A promotes non-small cell lung cancer progression by interacting with HuR and by promoting extracellular vesicles-mediated macrophage M2 polarization
Source: J Exp Clin Cancer Res. 2023 Dec 5;42:330. doi: 10.1186/s13046-023-02916-6 (PMC10696866; doi:10.1186/s13046-023-02916-6)
Supplement: Supplementary file 12 — Additional file 12: Table S3. Probes of FISH, RNA pull-down and ISH in this study. [file 13046_2023_2916_MOESM12_ESM.docx]

| Table S3. Probes of FISH, RNA pull-down and ISH in this study | |
| --- | --- |
| Probes | Sequence (5’-3’) |
| circATP9A FISH probe | TACTGGGTTCCCCTGGTGCTGCGAGT |
|  | 5’ cy3 |
| Biotin labeled control probe  (RNA pull-down) | CAGGAGTCTTCGTACTGCTTCTC  3' biotin |
| Biotin labeled circATP9A probe  (RNA pull-down) | GAGAAGCAGUACGAAGACUCCUG  3' biotin |
| circATP9A ISH probe | TGGTGCTGCGAGTGGCTGAGAT  5’-DIG labeled and 3’-DIG labeled |
| Scramble ISH probe (negative control) | GGGACCCGATGTATGGAGATAAGCA  5’-DIG labeled and 3’-DIG labeled |
| U6 ISH probe | AACGCTTCACGAATTTGCGT  5’ cy3 |

Abbreviation: DIG, Digoxigenin.
